# Impact of Cachexia and First‐Line Systemic Therapy for Previously Untreated Advanced Non‐Small Cell Lung Cancer: NEJ050A

**DOI:** 10.1002/jcsm.13606

**Published:** 2024-10-01

**Authors:** Keita Miura, Takehito Shukuya, Naoki Furuya, Ryo Morita, Akira Kisohara, Atsuto Mouri, Satoshi Watanabe, Hisashi Tanaka, Aya Hirata, Taiki Hakozaki, Kosuke Hamai, Naoko Matsumoto, Kana Watanabe, Hironori Ashinuma, Eisaku Miyauchi, Koji Sugano, Shinobu Hosokawa, Koji Amano, Satoshi Morita, Kunihiko Kobayashi, Makoto Maemonodo, Kazuhisa Takahashi

**Affiliations:** ^1^ Department of Respiratory Medicine Juntendo University Graduate School of Medicine Tokyo Japan; ^2^ Department of Internal Medicine, Division of Respiratory Medicine St Marianna University School of Medicine Kawasaki Japan; ^3^ Department of Respiratory Medicine Akita Kousei Medical Center Akita Japan; ^4^ Department of Respiratory Medicine Kasukabe Medical Center Saitama Japan; ^5^ Department of Respiratory Medicine, Comprehensive Cancer Center Saitama Medical University International Medical Center Hidaka Japan; ^6^ Department of Respiratory Medicine and Infectious Diseases Niigata University Graduate School of Medical and Dental Sciences Niigata Japan; ^7^ Department of Respiratory Medicine Hirosaki University Graduate School of Medicine Hirosaki Japan; ^8^ Department of Respiratory Medicine Kyorin University School of Medicine Tokyo Japan; ^9^ Department of Thoracic Oncology and Respiratory Medicine Tokyo Metropolitan Cancer and Infectious Diseases Center, Komagome Hospital Tokyo Japan; ^10^ Department of Respiratory Medicine Hiroshima Prefectural Hospital Hiroshima Japan; ^11^ Department of Respiratory Disease Hiroshima red Cross Hospital & Atomic‐Bomb Survivors Hospital Hiroshima Japan; ^12^ Department of Respiratory Medicine Miyagi Cancer Center Miyagi Japan; ^13^ Division of Respiratory Medicine Chiba Cancer Center Chiba Japan; ^14^ Department of Respiratory Medicine Tohoku University Hospital Sendai Japan; ^15^ Division of Respiratory Medicine Juntendo Tokyo Koto Geriatric Medical Center Tokyo Japan; ^16^ Department of Respiratory Medicine Japanese red Cross Okayama Hospital Okayama Japan; ^17^ Department of Supportive and Palliative Care Osaka International Cancer Institute Osaka Japan; ^18^ Department of Biomedical Statistics and Bioinformatics Kyoto University Graduate School of Medicine Kyoto Japan; ^19^ Department of Medicine, Division of Pulmonary Medicine Jichi Medical University Tochigi Japan

**Keywords:** anorexia, cancer cachexia, non‐small cell lung cancer, quality of life

## Abstract

**Background:**

Cancer cachexia complicates advanced non‐small cell lung cancer (NSCLC); however, it remains unclear how often cachexia occurs and how it affects the course of chemotherapy in patients receiving first‐line systemic therapy.

**Methods:**

We conducted a multicentre, prospective observational study and enrolled previously untreated NSCLC patients with Eastern Cooperative Oncology Group Performance Status (ECOG PS) of 0–2 and cachexia between September 2020 and September 2021. The primary outcome measure was the trends in the Functional Assessment of Anorexia/Cachexia Treatment and Anorexia/Cachexia Subscale [FAACT (A/CS)] scores by cohort. Secondary outcome measures included the incidence of cachexia before the initiation of first‐line systemic therapy, quality of life (QOL) measures, body weight (BW) changes, and efficacy and safety of first‐line systemic therapy.

**Results:**

A total of 887 consecutive patients with previously untreated advanced NSCLC and ECOG PS of 0–2 who were initiated on first‐line systemic therapy were evaluated. A total of 281 patients (31.7%) experienced BW loss consistent with the criteria of cachexia, and 186 were evaluated for QOL, BW and outcome measurements. Overall, 180/186 patients received first‐line systemic therapy. Cohort 1 (targeted therapy), cohort 2 [cytotoxic chemotherapy (CTx) ± immune checkpoint inhibitors (ICIs)] and cohort 3 (ICIs) included 42, 98 and 40 patients, respectively. There were significant variations in QOL trends by cohort, with chemotherapy‐associated emesis affecting early appetite‐related QOL. The change in the FAACT (A/CS) score at 1 week from baseline was worse in cohort 2 (the least square mean change ± standard error: −3.0 ± 0.9) than in cohorts 1 (1.6 ± 1.2, *p* = 0.003) and 3 (1.8 ± 1.0, *p* = 0.002); meanwhile, the change at 6 weeks was worse in cohort 1 (−1.5 ± 1.2) than in cohorts 2 (3.6 ± 0.9, *p* = 0.001) and 3 (3.5 ± 1.1, *p* = 0.004). BW reduction was observed in all cohorts within 6 weeks of therapy initiation. The targeted therapy cohort demonstrated superior progression‐free survival (PFS) and overall survival (OS) to CTx ± ICIs cohort or ICIs cohort (median PFS was 9.7 months, 6.3 months, 3.1 months, in cohort 1, 2, 3, respectively (cohort 1 vs. cohort 2: HR, 0.58, *p* = 0.018; cohort 1 vs. cohort 3: HR, 0.41, *p* = 0.001); median OS was not reached, 15.8 months, 9.9 months, respectively (cohort 1 vs. cohort 2: HR, 0.52, *p* = 0.033; cohort 1 vs. cohort 3: HR, 0.37, *p* = 0.003).

**Conclusions:**

Approximately 1/3 patients with previously untreated advanced NSCLC have cachexia. Appetite‐related QOL trends vary based on the type of first‐line systemic therapy in cachectic NSCLC patients, and the PFS and OS of these patients seemed to be shorter.

## 1 Introduction

Cancer cachexia is a multifactorial syndrome characterized by weight loss and anorexia that cannot be completely ameliorated by standard nutritional support alone [1]. This syndrome is driven primarily by systemic inflammation, leading to the breakdown of skeletal muscle and adipose tissue and resulting in decreased appetite [1]. Cachexia is highly common among patients with advanced lung cancer [2]. Weight loss in cancer patients, including those with non‐small cell lung cancer (NSCLC), is associated with a poor prognosis [3, 4].

Fearon et al. defined cancer cachexia as weight loss >5% within the last 6 months, body mass index (BMI) < 20 kg/m^2^ and any degree of weight loss >2%, or appendicular skeletal muscle index consistent with sarcopenia [1]. Of these, the definitions of body weight (BW) loss and BMI are a simple basis for screening and are often used as criteria for diagnosing cancer cachexia in clinical trials [5, 6, 7, 8]. The development of treatments for cachexia includes promising candidates such as ghrelin receptor agonists, selective androgen receptor modulators and anti‐inflammatory drugs [9, 10]. Moreover, a multidisciplinary approach combining pharmacological interventions with rehabilitation and nutritional support is being explored to manage cancer cachexia [11].

Novel therapies such as targeted therapy and immunotherapy have been developed since 2000, and these drugs showed superior efficacy compared with cytotoxic chemotherapy (CTx) [12, 13, 14, 15, 16, 17, 18, 19, 20]. Currently, targeted agents, CTx ± immune checkpoint inhibitors (ICIs), and ICIs are the standard treatment options for first‐line systemic therapy for advanced NSCLC. Cytotoxic agents are associated with a higher risk of anorexia than do targeted agents or ICIs [14, 15, 20]. Despite recent developments in antiemetic agents [21, 22, 23, 24], anorexia due to cytotoxic drugs remains a concern. As specific therapies for cancer cachexia are being developed, it is important to combine these therapies with drugs targeting the cancer itself, such as targeted agents, ICIs and CTx.

However, the impact of different first‐line systemic therapies on appetite‐related quality of life (QOL) and BW loss during treatment in previously untreated advanced NSCLC patients with cancer cachexia remains underexplored. To date, there is limited evidence regarding the presence of cachexia prior to initiating first‐line systemic therapy and its subsequent effects on QOL, weight loss and prognosis.

Therefore, in this study, we examine the incidence of cachexia in previously untreated advanced NSCLC patients and the change in QOL, weight loss and efficacy of first‐line systemic therapy, and conduct a prospective observational study of cachexia and first‐line systemic therapy for advanced NSCLC, in an exploratory approach.

## 2 Methods

### 2.1 Study Design and Participants

This multicentre, non‐interventional, prospective observational study enrolled patients from 33 sites in Japan between 11 September 2020 and 24 September 2021. Cachexic patients with previously untreated advanced NSCLC [postoperative recurrence or post curative intent (chemo) radiotherapy recurrence were also eligible] were eligible. Cachexia was defined as (i) weight loss >5% within the last 6 months and/or BMI < 20 kg/m^2^ and weight loss >2% within the last 6 months. The inclusion criteria were as follows: (1) age ≥20 years, (2) Eastern Cooperative Oncology Group (ECOG) Performance Status (PS) score of 0 to 2, and (3) scheduled to start first‐line systemic therapy. Patients who received corticosteroids were excluded. BW measured at 6 months before the initiation of systemic therapy at the hospital or at home was collected (self‐reported BW interviewed from the patients and written in the medical record was also acceptable).

The patients were enrolled before the start of first‐line systemic therapy and completed a questionnaire survey on QOL before and at 1, 3 and 6 weeks after the start of systemic therapy. To evaluate the incidence of cachexia in patients with previously untreated advanced NSCLC, we collected data from consecutive patients who received first‐line systemic therapy, met the above criteria of cachexia, and with an ECOG PS of 0–2. The patients were divided into three cohorts: cohort 1, patients who received targeted therapy; cohort 2, CTx ± ICIs; cohort 3, ICIs.

This study was approved by the Research Ethics Committee, Faculty of Medicine, Juntendo University (approval number: H20‐0177) and the ethics and institutional review boards of each participating institution and was conducted following the provisions of the Declaration of Helsinki [25]. Informed consent was obtained from the patients enrolled to this study. This study was registered in the University Medical Information Network Clinical Trials Registry (UMIN Clinical Trials Registry ID: UMIN000041813).

### 2.2 Outcome Measures

The primary outcome measures were the trends in Functional Assessment of Anorexia/Cachexia Treatment (FAACT), Anorexia/Cachexia Subscale (A/CS) score and appetite‐related QOL in each treatment cohort. The secondary outcome measures were the trends in the Questionnaire for Eating‐related Distress among Patients with Advanced Cancer (QERD) [26, 27] score, BW, efficacy and safety of first‐line systemic therapy, cachexia‐related laboratory values (haemoglobin, albumin and C‐reactive protein), and incidence of cachexia in previously untreated advanced NSCLC patients.

All QOL questionnaires used in this study were designed to assess cancer patients and self‐reported measures during the past 7 days. FAACT (A/CS) [28, 29] is a subscale of the FAACT [30, 31] that assesses the condition of a patient's appetite on a scale of 0 to 4, the higher the FAACT (A/CS) score, the higher QOL. QERD [26, 27] is specific to eating‐related distress and evaluates the condition on a scale of 1 to 5, the lower the QERD score, the higher QOL.

Responses to first‐line systemic therapy were evaluated according to the Response Evaluation Criteria in Solid Tumours version 1.1. [32] Progression‐free survival (PFS) was defined as the period between the start of first‐line systemic therapy and the date of progressive disease or death from any cause. Overall survival (OS) was defined as the period between the start of first‐line systemic therapy and the date of death from any cause. The safety of first‐line systemic therapy was investigated based on adverse events evaluated according to the Common Terminology Criteria for Adverse Events version 5.0.

### 2.3 Statistical Analysis

Analyses were performed based on a predetermined study protocol and statistical analysis plan. The full analysis set (FAS) was used to analyse the QOL score, ECOG PS, KPS, BW, safety and laboratory values. The FAS included patients whose QOL and BW were evaluated before the start of first‐line systemic therapy and observed at least once since the start of first‐line systemic therapy. The efficacy analysis set was used to analyse the efficacy of first‐line systemic therapy. This set involved all patients enrolled in this study and initiated first‐line systemic therapy.

We expected a case inclusion ratio of 3–4:5–6:1–2 for cohort 1: cohort 2: cohort 3, respectively. The mean (± standard deviation) FAACT (A/CS) score was 28–29 (8–9) points [29, 31], and the minimally important difference in FAACT (A/CS) score ranged from 1.70 to 6.95 [29]. Using a two‐sided 95% confidence interval (CI), a difference of 2.0, 2.5 and 3.0 in scores under a standard deviation of 8 could be evaluated in 62, 40 and 28 patients, respectively. Under a standard deviation of 9, the difference in scores of 2.5, 3.0 and 3.5 could be evaluated in 50, 35 and 26 patients, respectively. Considering that this study included the above three cohorts, we targeted to recruit 200 patients.

Descriptive statistics were used to summarize baseline parameters. The difference in the least‐squares (LS) mean from the initiation of first‐line systemic therapy to a specific time point was determined for each cohort. The difference in the mean LS between each cohort was compared using the analysis of variance. Categorical variables were compared using the Fisher's exact test. PFS and OS were evaluated using the Kaplan–Meier method and compared using the log‐rank test and Cox proportional hazards regression model. Descriptive statistics were used to assess safety parameters and reported as numbers and percentages. All statistical analyses were performed using EZR (Saitama Medical Center, Jichi Medical University, Saitama, Japan), a graphical user interface for R (R Foundation for Statistical Computing, Vienna, Austria). A *p*‐value of <0.05 was considered statistically significant.

## 3 Results

### 3.1 Incidence of Cachexia in Previously Untreated Advanced NSCLC

**FIGURE 1:**
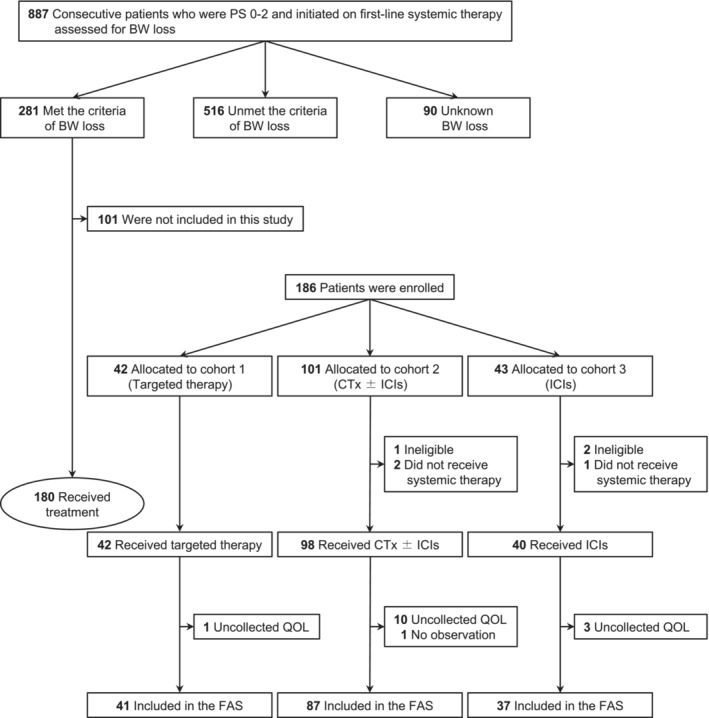
Study flow diagram. BW, body weight; CTx, cytotoxic chemotherapy; FAS, full analysis set; ICI, immune checkpoint inhibitor; QOL, quality of life.

All 887 consecutive patients with previously untreated advanced NSCLC and ECOG PS 0–2 were initiated on first‐line systemic therapy at the investigators' institutions during the study period (Figure 1). Among them, 281 patients (31.7%) had BW loss consistent with the criteria of cachexia, 516 (58.2%) did not have BW loss consistent with the above criteria, and 90 (10.1%) had unknown BW loss.

A total of 186 patients were enrolled in the study between September 2020 and September 2021, of which 180 received first‐line systemic therapy of targeted therapy (cohort 1, *n* = 42), CTx ± ICIs (cohort 2, *n* = 98) and ICIs (cohort 3, *n* = 40). These patients were included in the efficacy analysis set. There were 15 patients with uncollected QOL questionnaire or not evaluated, of whom 1 belonged to cohort 1; 11 to cohort 2, and 3 to cohort 3. The remaining 165 patients (cohort 1, *n* = 41, cohort 2, *n* = 87, and cohort 3, *n* = 37) were included in the FAS (Figure 1). The baseline characteristics of the three cohorts in the FAS are listed in Table 1. The median ages were 73, 70 and 77 years, and the median FAACT (A/CS) scores were 33, 31, and 28, in cohorts 1, 2, and 3, respectively. The details of the treatment regimens are shown in Tables S1 and S2. The median follow‐up period was 12.2 months (range, 0.1–21.6 months) in the efficacy analysis set.

**TABLE 1:** Patient characteristics by cohort in the FAS.

	Cohort 1 (*n* = 41)	Cohort 2 (*n* = 87)	Cohort 3 (*n* = 37)
Sex			
Male	15 (36.6)	65 (74.7)	31 (83.8)
Female	26 (63.4)	22 (25.3)	6 (16.2)
Age (years)	73 [43–88]	70 [42–86]	77 [58–87]
BW (kg)	50.1 ± 10.9	55.4 ± 11.9	54.4 ± 10.8
BW loss			
2%–5%	4 (9.8)	1 (1.1)	3 (8.1)
5%–10%	22 (53.7)	59 (67.8)	22 (59.5)
>10%	15 (36.6)	27 (31.0)	12 (32.4)
BMI (kg/m^2^)	21 ± 3.6	20.9 ± 3.4	20.3 ± 4.1
FAACT (A/CS)	33 ± 8.4	31 ± 8.1	28 ± 6.8
ECOG PS score			
0	8 (19.5)	12 (13.8)	4 (10.8)
1	22 (53.7)	60 (69.0)	23 (62.2)
2	11 (26.8)	15 (17.2)	10 (27.0)
Smoking history			
Yes	23 (56.1)	78 (89.7)	34 (91.9)
No	18 (43.9)	9 (10.3)	3 (8.1)
Disease stage (TNM 8th edition)			
IIIB/IIIC	0 (0.0)	2 (2.3)	1 (2.7)
IVA/IVB	36 (87.8)	71 (82.6)	30 (83.3)
Recurrence	5 (12.2)	14 (16.1)	6 (16.2)
Histology			
Adenocarcinoma	41 (100.0)	53 (60.9)	18 (48.6)
Squamous cell carcinoma	0 (0.0)	24 (27.6)	14 (37.8)
Other	0 (0.0)	10 (11.5)	5 (13.5)
Driver gene alteration			
*EGFR*	31 (75.6)	3 (3.4)	1 (2.7)
*ALK*	5 (12.2)	0 (0.0)	0 (0.0)
*ROS1*	2 (4.9)	0 (0.0)	0 (0.0)
*BRAF* V600E	2 (4.9)	0 (0.0)	0 (0.0)
*MET* exon 14 Skipping	1 (2.4)	0 (0.0)	0 (0.0)
PD‐L1 (Dako 22C3) TPS			
High positive (≥50%)	9 (22.0)	16 (18.4)	17 (45.9)
Low positive (1%–49%)	14 (34.1)	24 (27.6)	9 (24.3)
Negative (<1%)	12 (29.3)	29 (33.3)	7 (18.9)
Unknown	6 (14.6)	18 (20.7)	4 (10.8)

*Note:* Data are presented as no. (%), median ± SD, or median [range].

Abbreviations: BMI, body mass index; BW, body weight; CTx, cytotoxic chemotherapy; ECOG, Eastern Cooperative Oncology Group; FAACT (A/CS), Assessment of Anorexia/Cachexia Treatment Anorexia/Cachexia Subscale; FAS, full analysis set; ICIs, immune checkpoint inhibitors; PD‐L1, Programmed death‐ligand 1; PS, performance status; TPS, Tumour proportion score; SD, standard deviation.

### 3.2 QOL Questionnaire Score

**FIGURE 2:**
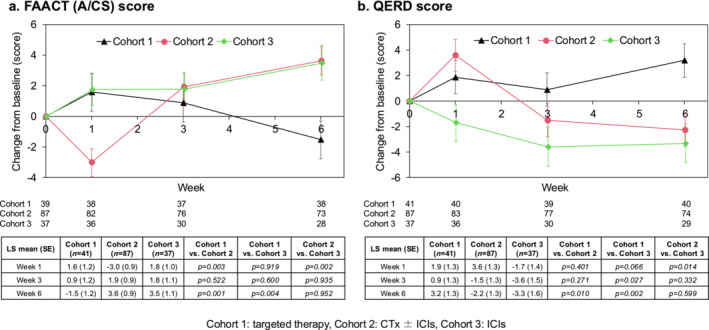
Time‐course change of QOL score. Line graph showing the trends in (a) FAACT (A/CS) score and (b) QERD score from baseline in each cohort in the FAS. The tables list the LS mean (SE) at 1, 3 and 6 weeks after initiating the systemic therapy and comparison among the cohorts using analysis of variance. CTx, cytotoxic chemotherapy; FAACT (A/CS), Functional Assessment of Anorexia/Cachexia Treatment Anorexia/Cachexia Subscale; FAS, full analysis set; ICI, immune checkpoint inhibitor; LS, least squares; QERD, Questionnaire for Eating‐related Distress among Patients with Advanced Cancer; QOL, quality of life; SE, standard error; BW, body weight.

The LS mean change (± standard error) in the FAACT (A/CS) score at 1 week from baseline in the FAS was 1.6 ± 1.2, −3.0 ± 0.9 and 1.8 ± 1.0 in cohorts 1, 2 and 3, respectively (Figure 2a). The change in the FAACT (A/CS) score was significantly worse in cohort 2 than cohorts 1 (*p* = 0.003) and 3 (*p* = 0.002). The LS mean change at 3 weeks from baseline was 0.9 ± 1.2, 1.9 ± 0.9 and 1.8 ± 1.1, and the scores were not significantly different in each cohort. The LS mean change at 6 weeks from baseline was −1.5 ± 1.2, 3.6 ± 0.9 and 3.5 ± 1.1, and the score was worse in cohort 1 than cohorts 2 (*p* = 0.001) and 3 (*p* = 0.004). The change in the QERD score at 1 week from baseline was significantly worse in cohort 2 than cohort 3 (*p* = 0.014), worse in cohort 1 than cohort 3 (*p* = 0.027) at 3 weeks and worse in cohort 1 than cohort 2 (*p* = 0.010) and 3 (*p* = 0.002) at 6 weeks (the LS mean change at 1 week from baseline: 1.9 ± 1.3, 3.6 ± 1.3, −1.7 ± 1.4; at 3 weeks: 0.9 ± 1.3, −1.5 ± 1.3, −3.6 ± 1.5; at 6 weeks: 3.2 ± 1.3, −2.2 ± 1.3, −3.3 ± 1.6) (Figure 2b). The QOL scores are shown in detail in Table S3.

The results of the univariate and multivariate analyses for the FAACT (A/CS) scores are shown in Table S4. In the univariate analysis, <75 and CTx ± ICIs treatment at 1 week, and female sex and targeted therapy at 6 weeks, were significantly associated with the deterioration of the FAACT (A/CS) score. Multivariate analysis showed that only CTx ± ICIs treatment was significantly associated with the deterioration of the FAACT (A/CS) score 1 week later.

### 3.3 Body Weight and Cachexia‐Related Laboratory Values

**FIGURE 3:**
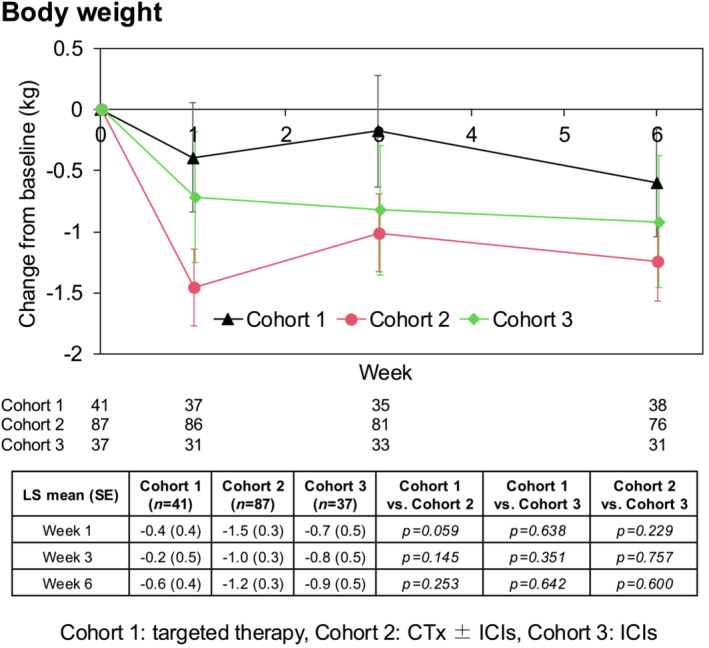
Time‐course change of BW. Line graph showing the trends in BW from baseline in each cohort in the FAS. The table lists the LS mean (SE) at 1, 3 and 6 weeks after initiating the systemic therapy and comparison among the cohorts using analysis of variance. CTx, cytotoxic chemotherapy; FAS, full analysis set; ICI, immune checkpoint inhibitor; LS, least squares; SE, standard error; BW, body weight.

The LS mean change in BW at 1 week from baseline in the FAS was −0.4 ± 0.4, −0.2 ± 0.5 and −0.6 ± 0.4, in cohorts 1, 2 and 3, respectively; at 3 week, −1.5 ± 0.3, −1.0 ± 0.3 and −1.2 ± 0.3; at 6 week, −0.7 ± 0.5, −0.8 ± 0.5 and −0.9 ± 0.5). The trends in the LS mean change in BW from baseline were not significantly different in each cohort in the FAS (Figure 3). However, the LS mean changes from baseline to 6 weeks after the start of systemic therapy were negative in all cohorts.

To clarify the relationship between appetite‐related QOL and BW gain, FAACT (A/CS) and QERD scores compared between the groups divided by the BW gain from baseline to maximum weight up to 6 weeks later (BW gain: ≤0% vs. >0%, ≤2.5% vs. >2.5% and ≤5.0% vs. >5.0%). In comparison among the three groups, the trends in the LS mean change in FAACT (A/CS) and QERD score from baseline were not significantly different. However, the groups that gained weight tended to have better appetite‐related QOL score (Figure S1).

Figure S2 shows transition of cachexia‐related laboratory values, that is, haemoglobin, albumin and C‐reactive protein. In cohort 2, the LS mean change in haemoglobin levels decreased over time at 1, 3 and 6 weeks from baseline, and furthermore, the change was significantly lower than in cohorts 1 and 3, at 3 and 6 weeks (*p* < 0.001). In albumin and C‐reactive protein levels, there were no significant differences in change from baseline at any time point. However, in albumin levels, all cohorts showed decreasing trends at 1 or 3 weeks from baseline, while recovering trends were observed at 6 weeks. In cohorts 2 and 3, the changes in C‐reactive protein levels from baseline increased at 1 week.

### 3.4 Survival Outcomes

**FIGURE 4:**
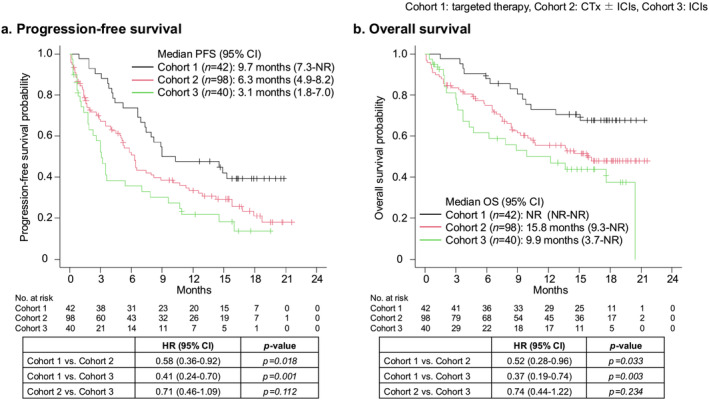
Efficacy of the first‐line systemic therapy. Kaplan–Meier survival curve of (a) PFS and (b) OS per cohort in the efficacy analysis set. The tables list the comparison among the cohort using the log‐rank test and Cox proportional hazards regression model. CTx, cytotoxic chemotherapy; HR, hazard ratio; ICI, immune checkpoint inhibitor; OS, overall survival; PFS, progression‐free survival.

The median PFS of first‐line systemic therapy in the efficacy analysis set was 9.7 months [95% confidence interval (CI), 7.3–not reached (NR)], 6.3 months (95% CI, 4.9–8.2) and 3.1 months (95% CI, 1.8–7.0) in cohorts 1, 2 and 3, respectively. Meanwhile, the median OS was NR (95% CI, NR–NR), 15.8 months (95% CI, 9.3‐NR) and 9.9 months (95% CI, 3.7‐NR) in cohorts 1, 2 and 3, respectively (Figure 4). Both PFS (cohort 1 vs. cohort 2: HR, 0.58 (95% CI, 0.36–0.92), *p* = 0.018; cohort 1 vs. cohort 3: HR, 0.41 (95% CI, 0.24–0.70), *p* = 0.001) and OS (cohort 1 vs. cohort 2: HR, 0.52 (95% CI, 0.28–0.96), *p* = 0.033; cohort 1 vs. cohort 3: HR, 0.37 (95% CI, 0.19–0.74, *p* = 0.003) significantly longer in cohort 1 than in cohorts 2 and 3.

The results of the univariate and multivariate analyses for the PFS and OS are shown in Table S5. Both in the univariate and multivariate analyses in PFS and OS, cohort 1 (targeted therapy) was independent better prognostic factor.

### 3.5 Frequency of Adverse Events

The treatment‐related adverse events in the FAS are shown in Table 2. The adverse event profiles differed in each cohort. Anorexia (any grade: 17.1%, 42.5% and 10.8%; grade ≥3:4.9%, 13.8%, and 2.7% in cohorts 1, 2 and 3, respectively) and nausea (any grade: 9.8%, 25.3%, and 2.7%; grade ≥3:0%, 4.6% and 0% in cohorts 1, 2 and 3, respectively) tended to occur more frequently in cohort 2. In contrast, diarrhoea (any grade: 39.0%, 14.9% and 2.7%; grade ≥3, 4.9%, 6.9% and 2.7% in cohorts 1, 2 and 3, respectively) and oral mucositis (oral mucositis: any grade, 17.1%, 6.9% and 0%; grade ≥3, 2.4%, 1.1% and 0% in cohorts 1, 2 and 3, respectively) tended to occur more frequently in cohort 1. Fatal treatment‐related adverse events occurred in two patients (2.3%) in cohort 2 (adrenal insufficiency and pneumonitis) and in one patient (2.7%) in cohort 3 (pneumonitis).

**TABLE 2:** Treatment‐related adverse events by cohort in the FAS.^a^

	Cohort 1 (*n* = 41)		Cohort 2 (*n* = 87)		Cohort 3 (*n* = 37)	
	All grades	Grade ≥3	All grade	Grade ≥3	All grade	Grade ≥3
Anaemia	5 (12.2)	0 (0.0)	45 (51.7)	15 (17.2)	2 (5.4)	0 (0.0)
Anorexia	7 (17.1)	2 (4.9)	37 (42.5)	12 (13.8)	4 (10.8)	1 (2.7)
White blood cell decreased	3 (7.3)	1 (2.4)	36 (41.4)	19 (21.8)	4 (10.8)	2 (5.4)
Alanine aminotransferase increased	7 (17.1)	2 (4.9)	25 (28.7)	5 (5.7)	6 (16.2)	2 (5.4)
Aspartate aminotransferase increased	8 (19.5)	2 (4.9)	19 (21.8)	5 (5.7)	6 (16.2)	3 (8.1)
Platelet count decreased	5 (12.2)	0 (0.0)	26 (29.9)	8 (9.2)	0 (0.0)	0 (0.0)
Diarrhoea	16 (39.0)	2 (4.9)	13 (14.9)	6 (6.9)	1 (2.7)	1 (2.7)
Malaise	0 (0.0)	0 (0.0)	25 (28.7)	5 (5.7)	5 (13.5)	2 (5.4)
Nausea	4 (9.8)	0 (0.0)	22 (25.3)	4 (4.6)	1 (2.7)	0 (0.0)
Constipation	3 (7.3)	0 (0.0)	18 (20.7)	1 (1.1)	1 (2.7)	0 (0.0)
Creatinine increased	2 (4.9)	0 (0.0)	17 (19.5)	4 (4.6)	3 (8.1)	1 (2.7)
Rash acneiform	8 (19.5)	0 (0.0)	7 (8.0)	0 (0.0)	3 (8.1)	0 (0.0)
Pneumonitis	4 (9.8)	1 (2.4)	7 (8.0)	4 (4.6)	5 (13.5)	2 (5.4)
Alopecia	0 (0.0)	0 (0.0)	12 (13.8)	0 (0.0)	3 (8.1)	0 (0.0)
Hyponatremia	3 (7.3)	0 (0.0)	11 (12.6)	1 (1.1)	1 (2.7)	0 (0.0)
Mucositis oral	7 (17.1)	1 (2.4)	6 (6.9)	1 (1.1)	0 (0.0)	0 (0.0)
Fever	0 (0.0)	0 (0.0)	9 (10.3)	0 (0.0)	3 (8.1)	0 (0.0)
Dry skin	8 (19.5)	0 (0.0)	4 (4.6)	1 (1.1)	0 (0.0)	0 (0.0)
Fatigue	0 (0.0)	0 (0.0)	8 (9.2)	1 (1.1)	3 (8.1)	0 (0.0)
Paronychia	10 (24.4)	0 (0.0)	0 (0.0)	0 (0.0)	0 (0.0)	0 (0.0)
Febrile neutropenia	0 (0.0)	0 (0.0)	9 (10.3)	9 (10.3)	0 (0.0)	0 (0.0)

*Note:* Data are presented as no. (%).

Abbreviations: CTx, cytotoxic chemotherapy; FAS, full analysis set; ICIs, immune checkpoint inhibitors.

^a^ The overall incidence of all‐grade adverse events (*n* = 165) is >5%.

## 4 Discussion

To the best of our knowledge, this study is the first to prospectively evaluate the incidence of cachexia before the start of first‐line systemic therapy in consecutive patients with previously untreated advanced NSCLC, and the impact of first‐line systemic therapy on appetite‐related QOL, BW and adverse events in patients with previously untreated advanced NSCLC with cachexia.

Approximately 1/3 of the PS 0–2 patients with previously untreated advanced NSCLC who received systemic therapy had BW loss consistent with the criteria of cachexia. The incidence of cachexia in previously untreated advanced NSCLC was similar to those previously reported in a large‐scale retrospective study [33, 34]. These findings are in line with the consensus published by the European Palliative Care Research Collaborative recommends the need for intervention from an earlier stage of cancer cachexia [1]. Patients should be appropriately evaluated for cachexia, and early intervention remains warranted.

The FAACT (A/CS) score was significantly worse at 1 week following initiation of first‐line systemic therapy in the patients who received CTx ± ICIs (cohort 2), and at 6 weeks in those who received targeted therapy (cohort 1). The reason for the deterioration in appetite‐related QOL after 1 week of systemic therapy could be the effect of acute and delayed emesis, as >90% of the patients in cohort 2 were treated with platinum‐based chemotherapy (Table S1). In this study, the incidence of anorexia in cohort 2 was higher than that in clinical trials of platinum‐based chemotherapy with or without ICIs [16, 17, 18, 19]. When patients with cachexia are treated with CTx ± ICIs, the management of anorexia should be carefully monitored.

The deteriorated appetite‐related QOL after 6 weeks in cohort 1 may be because the typical adverse side effects of epidermal growth factor receptor (EGFR) tyrosine kinase inhibitors (TKIs), such as diarrhoea and oral mucositis, could have negatively affected the appetite‐related QOL. More than 80% of the patients were treated with EGFR‐TKIs (Table S1). Diarrhoea and oral mucositis caused by EGFR‐TKIs tend to occur later than nausea and emesis caused by platinum‐based drugs, and this may explain why the appetite‐related QOL was only affected at 6 weeks after initiating first‐line systemic therapy.

In all cohorts, BW decreased for up to 6 weeks after the initiation of first‐line systemic therapy. The systemic treatment of patients with cachexia can potentially cause weight loss. Increased weight loss after initiating first‐line systemic therapy is associated with worse prognosis and QOL [35]. Thus, it is valuable to perform interventions, including exercise, nutritional support and drug treatment, for cachexia to maintain the general condition of patients with previously untreated advanced NSCLC with cachexia.

The PFS and OS were shorter in this study than those in clinical trials that did not consider the presence of cachexia [12, 15, 16]. In a large‐scale retrospective study in Japan, cancer cachexia was associated with a poor response to initial treatment in patients with advanced lung cancer, resulting in a poor prognosis [34], consistent with our findings. In patients with advanced NSCLC treated with PD‐1/PD‐L1 inhibitor monotherapy, the PFS and OS were significantly shorter in patients with cachexia [36]. A pharmacokinetic study of pembrolizumab for advanced NSCLC showed a shorter OS with rapid clearance of pembrolizumab [37]. Catabolic reactions due to cachexia may accelerate the clearance of pembrolizumab; therefore, cachexia may attenuate the efficacy of ICIs. In addition, the reason for the extremely poor PFS and OS in cohort 3 compared with previous reports may be related to a selection bias that the proportion of ECOG PS 2 patients was higher in cohort 3 than in cohort 2; thus, more patients avoided the administration of CTx.

Despite the fact that the treatment regimens were not standardized, especially in cohort 2, the frequency of anorexia was higher in this study than in the regulatory trials; on the other hand, no apparently more frequent adverse events were observed in each cohort [12, 13, 14, 15, 16, 17, 18, 19, 20]. In terms of management of adverse events, except for CTx‐induced anorexia, there may be no significant adverse events that would prevent the administration of first‐line systemic therapy for patients with previously untreated advanced NSCLC and cachexia with the PS of 0–2.

This study has some limitations. First, the extent to which appetite‐related QOL differs between patients with and without cachexia remains unclear because we evaluated QOL only in patients with cachexia. Second, supportive care management for patients with cachexia was not standardized and differed among institutions. Third, the criteria for cachexia only included weight loss; therefore, the study population included patients without anorexia or systemic inflammation. Fourth, although the primary and secondary endpoints were prespecified in this study, there was an exploratory component with no hypotheses regarding the transition in QOL; therefore, it is a limitation that the analyses are not corrected for multiplicity. Fifth, of the 15 QOL surveys that could not be collected, 11 were from cohort 2, and it is possible that the bias of missing QOL information may have affected the results. In addition, this study was typically a short follow‐up period, and more long‐term follow‐up is desirable. Despite these limitations, our findings provide novel insights into early intervention for advanced NSCLC with cancer cachexia, and these limitations should be addressed by future research.

## 5 Conclusions

This prospective observational study showed that appetite‐related QOL differed according to the type of first‐line systemic therapy among patients with previously untreated advanced NSCLC. In addition, BW decreased during the first‐line systemic therapy. Adverse events might be associated with appetite‐related QOL; therefore, management of adverse events is important for maintaining QOL. In this study, approximately one‐third of patients had BW loss consistent with the criteria for cachexia; the PFS and OS of these patients were shorter than those in previous clinical trials that did not consider the presence of cachexia. Attention should be paid to cancer cachexia screening, even before first‐line systemic therapy, in advanced or recurrent NSCLC. Future research remains warranted to develop effective therapeutic interventions for cancer cachexia.

## Conflicts of Interest

Keita Miura received honoraria from AstraZeneca K.K., Chugai Pharmaceutical Co., Ltd., Taiho Pharmaceutical Co., Ltd., and MSD K.K. outside the submitted work. Takehito Shukuya reports honoraria from AstraZeneca K.K., Chugai Pharmaceutical Co., Ltd., Nippon Boehringer Ingelheim Co., Ltd., Novartis Pharma K.K., and MSD K.K.; and honoraria from AstraZeneca K.K., Chugai Pharmaceutical Co., Ltd., Nippon Boehringer Ingelheim Co., Ltd., Novartis Pharma K.K., MSD K.K., Taiho Pharmaceutical Co., Ltd., DAIICHI SANKYO Co., Ltd., Ono Pharmaceutical Co., Ltd., Bristol‐Myers Squibb Company, Nippon Kayaku Co., Ltd., Takeda Pharmaceutical Company, Pfizer Inc., and Eli Lilly Japan K.K., outside the submitted work. Naoki Furuya reports honoraria from AstraZeneca K. K., Bristol‐Myers Squibb Company, Chugai Pharmaceutical Co., Ltd., Eli Lilly Japan K. K., and MSD K. K. outside of the submitted work. Ryo Morita received honoraria from AstraZeneca K.K., Bristol‐Myers Squibb Company, Amgen K.K., Novartis Pharma K.K., Takeda Pharmaceutical Company Limited, Daiichi Sankyo Co., Ltd., Chugai Pharmaceutical Co., Ltd., Eli Lilly Japan K.K., MSD K.K., Pfizer Inc., and Thermo Fisher Scientific Inc. outside of the submitted work. Atsuto Mouri reports honoraria from Chugai Pharmaceutical Co., Ltd. and Eli Lilly Japan K.K. outside of the submitted work. Satoshi Watanabe received honoraria from AstraZeneca K.K., Eli Lilly Japan K.K., Chugai Pharmaceutical Co., Ltd., Ono Pharmaceutical Co., Ltd., Taiho Pharmaceutical CO., LTD., Kyowa Kirin Co., Ltd., Takeda Pharmaceutical Company, Novartis Pharma K.K., Bristol‐Myers Squibb Company, Daiichi Sankyo Co., Ltd., Nippon Kayaku Co., Ltd., Merck Biopharma Co., Ltd., and Celltrion Healthcare Co., Ltd. outside the submitted work. Hisashi Tanaka reports honoraria from AstraZeneca K.K.; Bristol‐Myers Squibb Company; Chugai Pharmaceutical Co., Ltd.; Ono Pharmaceutical Co., Ltd.; Nippon Boehringer Ingelheim Co., Ltd.; Pfizer Inc.; and Takeda Pharmaceutical Company Limited. Taiki Hakozaki reports honoraria from Chugai Pharmaceutical Co., Ltd., Ono Pharmaceutical Co., Ltd., and Eisai Co., Ltd. outside of the submitted work. Hironori Ashinuma received honoraria from Ono Pharmaceutical Co., Ltd., Chugai Pharmaceutical Co., Ltd., Pfizer Inc., AstraZeneca K.K., Nippon Kayaku Co., Ltd., Bristol‐Myers Squibb Company, Merck Biopharma Co., Ltd., Eli Lilly Japan K.K., and Daiichi Sankyo Co., Ltd. outside the submitted work. Eisaku Miyauchi reports grants from Chugai Pharmaceutical Co., Ltd., and Eli Lilly Japan K.K.; honoraria from Taiho Pharmaceutical Co., Ltd., Daiichi Sankyo Co., Ltd., Nippon Boehringer Ingelheim Co., Ltd., Bristol‐Myers Squibb Company, Novartis Pharma K.K., MSD K.K., Kyowa Kirin Co., Ltd., Merck Biopharma Co., Ltd., Pfizer Inc., Ono Pharmaceutical Co., Ltd., Eisai Co., Ltd., Otsuka Pharmaceutical Co., Ltd., Chugai Pharmaceutical Co., Ltd., Amgen K.K., Thermo Fisher Scientific Inc., Nippon Kayaku Co., Ltd., Eli Lilly Japan K.K. Sysmex Co., Ltd., AstraZeneca K.K., and Kyorin Pharmaceutical Co., Ltd.,; and participation on the advisory board of Chugai Pharmaceutical Co., Ltd., Nippon Boehringer Ingelheim Co., Ltd., Eli Lilly Japan K.K. Merck Biopharma Co., Ltd., Daiichi Sankyo Co., Ltd., and Ono Pharmaceutical Co., Ltd., outside the submitted work. Satoshi Morita reports honoraria from AstraZeneca K.K.; Bristol‐Myers Squibb Company; Chugai Pharmaceutical Co., Ltd.; Eli Lilly Japan K.K.; MSD K.K.; Ono Pharmaceutical Co., Ltd.; and Pfizer Inc. outside of the submitted work. Kunihiko Kobayashi reports consulting fees from Daiichi Sankyo Co., Ltd. and reports honoraria from AstraZeneca K.K. and Takeda Pharmaceutical Company outside the submitted work. Makoto Maemondo reports honoraria from Ono Pharmaceutical Co., Ltd., MSD K.K., Bristol‐Myers Squibb Company, Chugai Pharmaceutical Co., Ltd., Taiho Pharmaceutical Co., Ltd., Nippon Boehringer Ingelheim Co., Ltd., Takeda Pharmaceutical Company Limited., and AstraZeneca K.K., outside the submitted work. Kazuhisa Takahashi reports grants from Chugai Pharmaceutical Co., Ltd., Nippon Boehringer Ingelheim Co., Ltd., Nippon Shinyaku Co., Ltd., Tsumura & Co., Pfizer Inc., Taiho Pharmaceutical Co., Ltd., Kyorin Pharmaceutical Co., Ltd, Teijin Pharma Limited, Sanofi K.K., Ono Pharmaceutical Co., Ltd., Novartis Pharma K.K., Shionogi & Co., Ltd., Eli Lilly Japan K.K., Bayer Yakuhin, Ltd, Daiichi Sankyo Co., Ltd., Nipro Pharma Corporation, Asahi Kasei Pharma Corporation, Nippon Kayaku Co., Ltd., Takeda Pharmaceutical Company Limited., and Kyowa Kirin Co., Ltd.; reports honoraria from Chugai Pharmaceutical Co., Ltd., Nippon Boehringer Ingelheim Co., Ltd., MSD K.K., Pfizer Inc., AstraZeneca K.K., Taiho Pharmaceutical Co., Ltd., Kyorin Pharmaceutical Co., LTD, Merck Biopharma Co., Ltd., Ono Pharmaceutical Co., Ltd., Nippon Kayaku Co., Ltd., Novartis Pharma K.K., Eli Lilly Japan K.K., Sumitomo Dainippon Pharma Co., Ltd., Bristol‐Myers Squibb Company, Meiji Seika Pharma Co., Ltd., Takeda Pharmaceutical Company Limited., Viatris Inc, Janssen Pharmaceutical K.K., Abbott Japan LLC, and Thermo Fisher Scientific Inc.; patent with Oncolys BioPharma Inc.; and leadership or fiduciary role with The Japan Lung Cancer Society and The Japan Respiratory Society, outside the submitted work. The other authors have no conflicts of interest to declare.

## Supporting information


**Table S1.** Details of the First‐Line Systemic Therapy (FAS)
**Table S2.** Details of the First‐Line Systemic Therapy (Efficacy Analysis Set)
**Table S3.** QOL Questionnaire Score
**Table S4.** Univariate and Multivariate Analysis for FAACT (A/CS) Score
**Table S5.** Univariate and Multivariate Analysis for Survival Outcomes

**Figure S1.** Relationship between appetite‐related QOL and BW gain. Line graph showing the trends in FAACT (A/CS) score and QERD score from baseline between the groups divided by the BW gain from baseline to maximum weight up to 6 weeks later (BW gain: (a, b) ≤ 0% vs. >0%, (c, d) ≤ 2.5% vs. >2.5% and (e, f) ≤ 5.0% vs. >5.0%) in the FAS. The tables list showed the QOL scores in detail. The LS mean (SE) at 1, 3, and 6 weeks after initiating the systemic therapy and comparison between the groups using analysis of variance Abbreviations: BW, body weight, FAACT (A/CS), Functional Assessment of Anorexia/Cachexia Treatment Anorexia/Cachexia Subscale; FAS, full analysis set; LS, least squares; QERD, Questionnaire for Eating‐related Distress among Patients with Advanced Cancer; QOL, quality of life; SD, standard deviation; SE, standard error
**Figure S2.** Time‐course change of cachexia‐related laboratory values. Line graph showing the trends in (a) haemoglobin, (b) albumin and (c) c‐reactive protein levels from baseline in each cohort in the FAS. The table lists the LS mean (SE) at 1, 3, and 6 weeks after initiating the systemic therapy and comparison among the cohorts using analysis of variance Abbreviations:CTx, cytotoxic chemotherapy; FAS, full analysis set; ICI, immune checkpoint inhibitor; LS, least squares; SE, standard error
