# Supplementary material for: Impact of Cachexia and First‐Line Systemic Therapy for Previously Untreated Advanced Non‐Small Cell Lung Cancer: NEJ050A
Source: J Cachexia Sarcopenia Muscle. 2024 Oct 1;15(6):2618–28. doi: 10.1002/jcsm.13606 (PMC11634491; doi:10.1002/jcsm.13606)
Supplement: Supplementary file 1 — Table S1. Details of the First‐Line Systemic Therapy (FAS) Table S2. Details of the First‐Line Systemic Therapy (Efficacy Analysis Set) Table S3. QOL Questionnaire Score Table S4. Univariate and Multivariate Analysis for FAACT (A/CS) Score Table S5. Univariate and Multivariate Analysis for Survival Outcomes [file JCSM-15-2618-s002.docx]

**Impact of cachexia and first-line systemic therapy for previously untreated advanced non-small cell lung cancer: NEJ050A**

Journal name: The Journal of Cachexia, Sarcopenia and Muscle

Keita Miura, MD^1^; Takehito Shukuya, MD, PhD^1^; Naoki Furuya, MD, PhD^2^; Ryo Morita, MD, PhD^3^; Akira Kisohara, MD, PhD^4^; Atsuto Mouri, MD, PhD^5^; Satoshi Watanabe, MD, PhD^6^; Hisashi Tanaka, MD, PhD^7^; Aya Hirata, MD, PhD^8^; Taiki Hakozaki, MD^9^; Kosuke Hamai, MD, PhD^10^; Naoko Matsumoto, MD^11^; Kana Watanabe, MD^12^; Hironori Ashinuma, MD, PhD^13^; Eisaku Miyauchi, MD, PhD^14^; Koji Sugano, MD, PhD^15^; Shinobu Hosokawa, MD, PhD^16^; Koji Amano, MD, PhD^17^; Satoshi Morita, PhD^18^; Kunihiko Kobayashi, MD, PhD^5^; Makoto Maemonodo, MD, PhD^19^; Kazuhisa Takahashi, MD, PhD^1^

^1^Department of Respiratory Medicine, Juntendo University Graduate School of Medicine, Tokyo, Japan

^2^Division of Respiratory Medicine, Department of Internal Medicine, St Marianna University School of Medicine, Kanagawa, Japan

^3^Department of Respiratory Medicine, Akita Kousei Medical Center, Akita, Japan

^4^Department of Respiratory Medicine, Kasukabe Medical Center, Saitama, Japan

^5^Department of Respiratory Medicine, Comprehensive Cancer Center, Saitama Medical University International Medical Center, Hidaka, Japan

^6^Department of Respiratory Medicine and Infectious Diseases, Niigata University Graduate School of Medical and Dental Sciences, Niigata, Japan

^7^Department of Respiratory Medicine, Hirosaki University Graduate School of Medicine, Hirosaki, Japan

^8^Department of Respiratory Medicine, Kyorin University School of Medicine, Tokyo, Japan

^9^Department of Thoracic Oncology and Respiratory Medicine, Tokyo Metropolitan Cancer and Infectious Diseases Center, Komagome Hospital, Tokyo, Japan

^10^Department of Respiratory Medicine, Hiroshima Prefectural Hospital, Hiroshima, Japan

^11^Department of Respiratory Disease, Hiroshima Red Cross Hospital & Atomic-Bomb Survivors Hospital, Hiroshima, Japan

^12^Department of Respiratory Medicine, Miyagi Cancer Center, Miyagi, Japan.

^13^Division of Respiratory Medicine, Chiba Cancer Center, Chiba, Japan

^14^Department of Respiratory Medicine, Tohoku University Hospital, Miyagi, Japan

^15^Division of Respiratory Medicine, Juntendo Tokyo Koto Geriatric Medical Center, Tokyo, Japan

^16^Department of Respiratory Medicine, Japanese Red Cross Okayama Hospital, Okayama, Japan

^17^Department of Supportive and Palliative Care, Osaka International Cancer Institute, Osaka, Japan

^18^Department of Biomedical Statistics and Bioinformatics, Kyoto University Graduate School of Medicine, Kyoto, Japan

^19^Division of Pulmonary Medicine, Department of Medicine, Jichi Medical University, Tochigi, Japan

**Address correspondence and reprint requests to**

Takehito Shukuya, MD, PhD

Department of Respiratory Medicine, Juntendo University Graduate School of Medicine, 3-1-3 Hongo, Bunkyo-ku, Tokyo 113-8421, Japan

Tel: +81-3-5802-1063, Fax: +81-3-5802-1617

E-mail: tshukuya@juntendo.ac.jp

**Table S1. Details of the First-Line Systemic Therapy (FAS)**

| **Regimen** | **No. (%)** |
| --- | --- |
| **Cohort 1 (targeted therapy)** | **41** |
| Osimertinib | 27 (65.9) |
| Afatinib | 7 (17.1) |
| Dabrafenib + Trametinib | 2 (4.9) |
| Gefitinib | 1 (2.4) |
| Crizotinib | 1 (2.4) |
| Brigatinib | 1 (2.4) |
| Tepotinib | 1 (2.4) |
| Entrectinib | 1 (2.4) |
| **Cohort 2 (cytotoxic chemotherapy ± ICIs)** | **87** |
| Cytotoxic chemotherapy + ICIs | 63 (72.4) |
| CBDCA + nab-PTX + Pembrolizumab | 13 (14.9) |
| CBDCA + PEM + Pembrolizumab | 9 (10.3) |
| CBDCA + PEM + Nivolumab + Ipilimumab | 9 (10.3) |
| CBDCA + PTX + Nivolumab + Ipilimumab | 9 (10.3) |
| CBDCA + nab-PTX + Atezolizumab | 8 (9.2) |
| CBDCA + PEM + Atezolizumab | 4 (4.6) |
| CDDP + PEM + Nivolumab + Ipilimumab | 4 (4.6) |
| CBDCA + PTX + Bev + Atezolizumab | 3 (3.4) |
| CDDP + PEM + Pembrolizumab | 1 (1.1) |
| PEM + Pembrolizumab | 1 (1.1) |
| CBDCA + ETP + Atezolizumab | 1 (1.1) |
| CDDP + ETP + Durvalumab | 1 (1.1) |
| Cytotoxic chemotherapy | 24 (23.0) |
| CBDCA + nab-PTX | 7 (8.0) |
| CBDCA + PEM | 3 (3.4) |
| CBDCA + PEM + Bev | 3 (3.4) |
| CBDCA + S-1 | 2 (2.3) |
| CBDCA + ETP | 2 (2.3) |
| CDDP + PEM | 1 (1.1) |
| CBDCA + PTX | 1 (1.1) |
| CBDCA + PTX + Bev | 1 (1.1) |
| CDDP + ETP | 1 (1.1) |
| PEM | 1 (1.1) |
| S-1 | 1 (1.1) |
| CPT-11 | 1 (1.1) |
| **Cohort 3 (ICIs)** | **37** |
| Pembrolizumab | 22 (59.5) |
| Nivolumab + Ipilimumab | 15 (40.5) |

Data are presented as No. (%).

Bev, bevacizumab; CBDCA, carboplatin; CDDP, cisplatin; CPT-11, irinotecan; ETP, etoposide; FAS, full analysis set; ICI, immune checkpoint inhibitor; nab-PTX, nanoparticle albumin-bound paclitaxel; PEM, pemetrexed; PTX, paclitaxel; S-1, tegafur/gimeracil/oteracil

**Table S2. Details of the First-Line Systemic Therapy (Efficacy Analysis Set)**

| **Regimen** | **No. (%)** |
| --- | --- |
| **Cohort 1 (targeted therapy)** | **42** |
| Osimertinib | 28 (66.7) |
| Afatinib | 7 (16.7) |
| Dabrafenib + Trametinib | 2 (4.8) |
| Gefitinib | 1 (2.4) |
| Crizotinib | 1 (2.4) |
| Brigatinib | 1 (2.4) |
| Tepotinib | 1 (2.4) |
| Entrectinib | 1 (2.4) |
| **Cohort 2 (cytotoxic chemotherapy ± ICIs)** | **98** |
| Cytotoxic chemotherapy + ICIs | 73 (73.7) |
| CBDCA + nab-PTX + Pembrolizumab | 15 (15.3) |
| CBDCA + PEM + Nivolumab + Ipilimumab | 11 (11.2) |
| CBDCA + nab-PTX + Atezolizumab | 10 (10.2) |
| CBDCA + PTX + Nivolumab + Ipilimumab | 10 (10.2) |
| CBDCA + PEM + Pembrolizumab | 9 (9.2) |
| CBDCA + PEM + Atezolizumab | 7 (7.1) |
| CDDP + PEM + Nivolumab + Ipilimumab | 4 (4.1) |
| CBDCA + PTX + Bev + Atezolizumab | 3 (3.1) |
| CDDP + PEM + Pembrolizumab | 1 (1.0) |
| PEM + Pembrolizumab | 1 (1.0) |
| CBDCA + ETP + Atezolizumab | 1 (1.0) |
| CDDP + ETP + Durvalumab | 1 (1.0) |
| Cytotoxic chemotherapy | 25 (24.0) |
| CBDCA + nab-PTX | 7 (7.1) |
| CBDCA + PEM | 3 (3.1) |
| CBDCA + PEM + Bev | 3 (3.1) |
| CBDCA + S-1 | 3 (3.1) |
| CBDCA + ETP | 2 (2.0) |
| CDDP + PEM | 1 (1.0) |
| CBDCA + PTX | 1 (1.0) |
| CBDCA + PTX + Bev | 1 (1.0) |
| CDDP + ETP | 1 (1.0) |
| PEM | 1 (1.0) |
| S-1 | 1 (1.0) |
| CPT-11 | 1 (1.0) |
| **Cohort 3 (ICIs)** | **40** |
| Pembrolizumab | 23 (57.5) |
| Nivolumab + Ipilimumab | 17 (42.5) |

Data are presented as No. (%).

Bev, bevacizumab; CBDCA, carboplatin; CDDP, cisplatin; CPT-11, irinotecan; ETP, etoposide; ICI, immune checkpoint inhibitor; nab-PTX, nanoparticle albumin-bound paclitaxel; PEM, pemetrexed; PTX, paclitaxel; S-1, tegafur/gimeracil/oteracil

**Table S3. QOL Questionnaire Score**

**Table S3a.** FAACT (A/CS) Score

|  | No. | Mean | SD | SE | minimum | 25th quartile | Median | 75th percentile | maximum |
| --- | --- | --- | --- | --- | --- | --- | --- | --- | --- |
| Baseline |  |  |  |  |  |  |  |  |  |
| Cohort 1 (targeted therapy) | 39 | 31.2 | 8.4 | 1.3 | 14 | 25 | 33 | 37.5 | 44 |
| Cohort 2 (CTx ± ICIs) | 87 | 30.1 | 8.1 | 0.9 | 9 | 24 | 31 | 35 | 44 |
| Cohort 3 (ICIs) | 37 | 29.4 | 6.8 | 1.1 | 20 | 26 | 28 | 33 | 45 |
| Week 1 |  |  |  |  |  |  |  |  |  |
| Cohort 1 (targeted therapy) | 40 | 33.0 | 8.3 | 1.3 | 9 | 28.4 | 34.5 | 38 | 46 |
| Cohort 2 (CTx ± ICIs) | 82 | 27.3 | 8.7 | 1.0 | 7 | 21 | 28 | 33.6 | 46 |
| Cohort 3 (ICIs) | 36 | 31.2 | 6.7 | 1.1 | 14 | 27.8 | 31 | 35.25 | 44 |
| Week 3 |  |  |  |  |  |  |  |  |  |
| Cohort 1 (targeted therapy) | 39 | 32.6 | 6.8 | 1.1 | 17 | 29 | 32 | 36.5 | 47 |
| Cohort 2 (CTx ± ICIs) | 76 | 32.3 | 7.7 | 0.9 | 10 | 28.8 | 32 | 38 | 46 |
| Cohort 3 (ICIs) | 30 | 31.5 | 7.7 | 1.4 | 14 | 27 | 32.5 | 36 | 43 |
| Week 6 |  |  |  |  |  |  |  |  |  |
| Cohort 1 (targeted therapy) | 39 | 30.0 | 9.4 | 1.5 | 10 | 22 | 31 | 36 | 45 |
| Cohort 2 (CTx ± ICIs) | 73 | 34.4 | 7.9 | 0.9 | 6 | 30 | 36 | 40 | 48 |
| Cohort 3 (ICIs) | 28 | 33.3 | 7.1 | 1.3 | 22 | 27 | 34 | 38.3 | 47 |

FAACT (A/CS), Assessment of Anorexia/Cachexia Treatment Anorexia/Cachexia Subscale; SD, standard deviation; SE standard error

**Table S3b.** Time-Course Change of FAACT (A/CS) Score from Baseline

|  | No. | Mean | SD | SE | minimum | 25th quartile | Median | 75th percentile | maximum | LS mean | SE in LS mean |
| --- | --- | --- | --- | --- | --- | --- | --- | --- | --- | --- | --- |
| Week 1 |  |  |  |  |  |  |  |  |  |  |  |
| Cohort 1 (targeted therapy) | 38 | 1.6 | 7.2 | 1.2 | -17 | -3 | 2 | 6 | 20 | 1.6 | 1.2 |
| Cohort 2 (CTx ± ICIs) | 82 | -3.1 | 8.3 | 0.9 | -28 | -7 | -1.8 | 3 | 13 | -3.0 | 0.9 |
| Cohort 3 (ICIs) | 36 | 1.8 | 5.3 | 0.9 | -11 | -2.1 | 2 | 5 | 10 | 1.8 | 1.0 |
| Week 3 |  |  |  |  |  |  |  |  |  |  |  |
| Cohort 1 (targeted therapy) | 37 | 1.2 | 7.5 | 1.2 | -18 | -2 | 1 | 8 | 15 | 0.9 | 1.2 |
| Cohort 2 (CTx ± ICIs) | 76 | 1.7 | 7.0 | 0.8 | -26 | -2 | 2.3 | 4.3 | 19 | 1.9 | 0.9 |
| Cohort 3 (ICIs) | 30 | 1.5 | 7.4 | 1.4 | -16 | -2.3 | 1.5 | 6 | 19 | 1.8 | 1.1 |
| Week 6 |  |  |  |  |  |  |  |  |  |  |  |
| Cohort 1 (targeted therapy) | 38 | -1.5 | 9.2 | 1.5 | -26 | -6.8 | -0.5 | 5.3 | 14 | -1.5 | 1.2 |
| Cohort 2 (CTx ± ICIs) | 73 | 3.2 | 8.8 | 1.0 | -24 | -1 | 3 | 9 | 28 | 3.6 | 0.9 |
| Cohort 3 (ICIs) | 28 | 3.1 | 5.9 | 1.1 | -8 | -1.8 | 3 | 6.5 | 17 | 3.5 | 1.1 |

FAACT (A/CS), Assessment of Anorexia/Cachexia Treatment Anorexia/Cachexia Subscale; LS, least square; SD, standard deviation; SE standard error

**Table S3c.** QERD Score

|  | No. | Mean | SD | SE | minimum | 25th quartile | Median | 75th percentile | maximum |
| --- | --- | --- | --- | --- | --- | --- | --- | --- | --- |
| Baseline |  |  |  |  |  |  |  |  |  |
| Cohort 1 (targeted therapy) | 41 | 24.8 | 13.0 | 2.0 | 12 | 13 | 19 | 35 | 57 |
| Cohort 2 (CTx ± ICIs) | 87 | 28.3 | 11.9 | 1.3 | 12 | 17 | 28 | 37 | 60 |
| Cohort 3 (ICIs) | 37 | 29.8 | 11.8 | 1.9 | 12 | 21.8 | 31 | 40 | 49 |
| Week 1 |  |  |  |  |  |  |  |  |  |
| Cohort 1 (targeted therapy) | 40 | 26.6 | 12.4 | 2.0 | 12 | 13 | 26 | 37 | 53 |
| Cohort 2 (CTx ± ICIs) | 83 | 31.8 | 11.6 | 1.3 | 12 | 24 | 32 | 41 | 59 |
| Cohort 3 (ICIs) | 36 | 27.9 | 10.5 | 1.8 | 12 | 22.3 | 28 | 34.3 | 56 |
| Week 3 |  |  |  |  |  |  |  |  |  |
| Cohort 1 (targeted therapy) | 39 | 25.2 | 11.9 | 1.9 | 12 | 12 | 24 | 34 | 49 |
| Cohort 2 (CTx ± ICIs) | 77 | 26.5 | 10.6 | 1.2 | 12 | 17 | 27 | 34 | 60 |
| Cohort 3 (ICIs) | 30 | 25.6 | 11.5 | 2.1 | 12 | 14 | 26.5 | 33 | 52 |
| Week 6 |  |  |  |  |  |  |  |  |  |
| Cohort 1 (targeted therapy) | 40 | 27.9 | 11.4 | 1.8 | 12 | 18.3 | 28 | 35.3 | 51 |
| Cohort 2 (CTx ± ICIs) | 74 | 25.7 | 12.0 | 1.4 | 12 | 16 | 24 | 33 | 60 |
| Cohort 3 (ICIs) | 29 | 25.7 | 11.3 | 2.1 | 12 | 15 | 25 | 35 | 49 |

QERD, Questionnaire for Eating-related Distress among Patients with Advanced Cancer; SD, standard deviation; SE standard error

**Table S3d.** Time-Course Change of QERD Score from Baseline

|  | No. | Mean | SD | SE | minimum | 25th quartile | Median | 75th percentile | maximum | LS mean | SE in LS mean |
| --- | --- | --- | --- | --- | --- | --- | --- | --- | --- | --- | --- |
| Week 1 |  |  |  |  |  |  |  |  |  |  |  |
| Cohort 1 (targeted therapy) | 40 | 1.5 | 8.1 | 1.3 | -23 | -1 | 0 | 3.8 | 18 | 1.9 | 1.3 |
| Cohort 2 (CTx ± ICIs) | 83 | 3.7 | 11.7 | 1.3 | -18 | -4.5 | 2 | 11.5 | 35 | 3.6 | 1.3 |
| Cohort 3 (ICIs) | 36 | -1.6 | 8.4 | 1.4 | -24 | -5 | -1.5 | 1.8 | 17 | -1.7 | 1.4 |
| Week 3 |  |  |  |  |  |  |  |  |  |  |  |
| Cohort 1 (targeted therapy) | 39 | 0.6 | 7.8 | 1.3 | -18 | -2 | 0 | 2.6 | 19 | 0.9 | 1.3 |
| Cohort 2 (CTx ± ICIs) | 77 | -1.1 | 10.8 | 1.2 | -25 | -8 | -1 | 2 | 37 | -1.5 | 1.3 |
| Cohort 3 (ICIs) | 30 | -2.9 | 9.5 | 1.7 | -24.5 | -7.8 | -2.5 | 0 | 21 | -3.6 | 1.5 |
| Week 6 |  |  |  |  |  |  |  |  |  |  |  |
| Cohort 1 (targeted therapy) | 40 | 3.0 | 9.9 | 1.6 | -18 | -3.3 | 2.5 | 8.3 | 32 | 3.2 | 1.3 |
| Cohort 2 (CTx ± ICIs) | 74 | -2.2 | 11.8 | 1.4 | -27 | -9 | -2 | 3 | 40 | -2.2 | 1.3 |
| Cohort 3 (ICIs) | 29 | -3.0 | 7.6 | 1.4 | -17 | -7 | -2.5 | 1 | 16 | -3.3 | 1.6 |

LS, least square; QERD, Questionnaire for Eating-related Distress among Patients with Advanced Cancer; SD, standard deviation; SE standard error

**Table S4. Univariate and Multivariate Analysis for FAACT (A/CS) Score**

**Table S4a.** At 1 Week after the Start of First-Line Systemic Therapy

|  | Regression coefficient (95% confidence interval) | | | |
| --- | --- | --- | --- | --- |
|  | Univariate analysis | *p*-value | Multivariate analysis | *p*-value |
| Sex |  |  |  |  |
| Male | 0 |  | 0 |  |
| Female | 0.757 (-1.860-3.374) | *0.569* | -0.034 (-2.801-2.733) | *0.981* |
| Age |  |  |  |  |
| <75 | 0 |  | 0 |  |
| ≥75 | 2.979 (0.512-5.445) | *0.018* | 1.981 (-0.523-4.484) | *0.120* |
| BW loss |  |  |  |  |
| <10% | 0 |  | 0 |  |
| ≥10% | 1.452 (-1.090-3.994) | *0.261* | 1.119 (-1.415-3.654) | *0.384* |
| BMI |  |  |  |  |
| <20 | 0 |  | 0 |  |
| ≥20 | -0.467 (-2.978-2.043) | *0.714* | 0.043 (-2.491-2.577) | *0.973* |
| ECOG PS score |  |  |  |  |
| 0-1 | 0 |  | 0 |  |
| 2 | 2.171 (-0.853-5.196) | *0.158* | 1.149 (-1.821-4.119) | *0.446* |
| Cohort |  |  |  |  |
| Cohort 1 (targeted therapy) | -0.194 (-3.604-3.216) | *0.911* | -0.025 (-3.766-3.716) | *0.989* |
| Cohort 2 (CTx ± ICIs) | -4.839 (-7.77--1.908) | *0.001* | -4.124 (-7.234--1.015) | *0.010* |
| Cohort 3 (ICIs) | 0 |  | 0 |  |

Data are presented as regression coefficient (95% confidence interval).

BMI, body mass index; BW, body weight; CTx, cytotoxic chemotherapy; ECOG, Eastern Cooperative Oncology Group, FAACT (A/CS), Assessment of Anorexia/Cachexia Treatment Anorexia/Cachexia Subscale; ICI, immune checkpoint inhibitor; PS, performance status

**Table S4b.** At 3 Weeks after the Start of First-Line Systemic Therapy

|  | Regression coefficient (95% confidence interval) | | | |
| --- | --- | --- | --- | --- |
|  | Univariate analysis | *p*-value | Multivariate analysis | *p*-value |
| Sex |  |  |  |  |
| Male | 0 |  | 0 |  |
| Female | 0.076 (-2.440-2.591) | *0.953* | 0.066 (-2.759-2.892) | *0.963* |
| Age |  |  |  |  |
| <75 | 0 |  | 0 |  |
| ≥75 | 0.532 (-1.912-2.976) | *0.668* | 0.493 (-2.084-3.071) | *0.706* |
| BW loss |  |  |  |  |
| <10% | 0 |  | 0 |  |
| ≥10% | 1.776 (-0.699-4.250) | *0.158* | 1.732 (-0.855-4.320) | *0.188* |
| BMI |  |  |  |  |
| <20 | 0 |  | 0 |  |
| ≥20 | 0.391 (-2.062-2.844) | *0.753* | 0.725 (-1.837-3.287) | *0.576* |
| ECOG PS score |  |  |  |  |
| 0-1 | 0 |  | 0 |  |
| 2 | 1.689 (-1.441-4.820) | *0.288* | 1.660 (-1.632-4.952) | *0.320* |
| Cohort |  |  |  |  |
| Cohort 1 (targeted therapy) | -0.276 (-3.795-3.243) | *0.877* | -0.543 (-4.422-3.336) | *0.782* |
| Cohort 2 (CTx ± ICIs) | 0.154 (-2.934-3.242) | *0.922* | 0.411 (-2.904-3.725) | *0.807* |
| Cohort 3 (ICIs) | 0 |  | 0 |  |

Data are presented as regression coefficient (95% confidence interval).

BMI, body mass index; BW, body weight; CTx, cytotoxic chemotherapy; ECOG, Eastern Cooperative Oncology Group, FAACT (A/CS), Assessment of Anorexia/Cachexia Treatment Anorexia/Cachexia Subscale; ICI, immune checkpoint inhibitor; PS, performance status

**Table S4c.** At 6 Weeks After the Start of First-Line Systemic Therapy

|  | Regression coefficient (95% confidence interval) | | | |
| --- | --- | --- | --- | --- |
|  | Univariate analysis | *p*-value | Multivariate analysis | *p*-value |
| Sex |  |  |  |  |
| Male | 0 |  | 0 |  |
| Female | -3.535 (-6.561--0.508) | *0.022* | -1.832 (-5.265-1.602) | *0.293* |
| Age |  |  |  |  |
| <75 | 0 |  | 0 |  |
| ≥75 | -1.807 (-4.759-1.146) | *0.228* | -1.429 (-4.458-1.599) | *0.352* |
| BW loss |  |  |  |  |
| <10% | 0 |  | 0 |  |
| ≥10% | 0.427 (-2.572-3.426) | *0.779* | 1.094 (-1.937-4.125) | *0.476* |
| BMI |  |  |  |  |
| <20 | 0 |  | 0 |  |
| ≥20 | 0.998 (-1.997-3.993) | *0.511* | 1.149 (-1.927-4.225) | *0.461* |
| ECOG PS score |  |  |  |  |
| 0-1 | 0 |  | 0 |  |
| 2 | -0.061 (-3.967-3.845) | *0.975* | 0.75 (-3.180-4.680) | *0.706* |
| Cohort |  |  |  |  |
| Cohort 1 (targeted therapy) | -4.655 (-8.803--0.508) | *0.028* | -4.044 (-8.699-0.611) | *0.088* |
| Cohort 2 (CTx ± ICIs) | 0.095 (-3.607-3.796) | *0.960* | -0.163 (-4.115-3.790) | *0.935* |
| Cohort 3 (ICIs) | 0 |  | 0 |  |

Data are presented as regression coefficient (95% confidence interval).

BMI, body mass index; BW, body weight; CTx, cytotoxic chemotherapy; ECOG, Eastern Cooperative Oncology Group, FAACT (A/CS), Assessment of Anorexia/Cachexia Treatment Anorexia/Cachexia Subscale; ICI, immune checkpoint inhibitor; PS, performance status

**Table S5. Univariate and Multivariate Analysis for Survival Outcomes**

**Table S5a.** Univariate and Multivariate Analysis for PFS

|  | Hazard ratio (95% confidence interval) | | | |
| --- | --- | --- | --- | --- |
|  | Univariate analysis | *p*-value | Multivariate analysis | *p*-value |
| Sex |  |  |  |  |
| Male | 1 |  | 1 |  |
| Female | 0.633 (0.428-0.936) | *0.022* | 0.768 (0.504-1.17) | *0.219* |
| Age |  |  |  |  |
| <75 | 1 |  | 1 |  |
| ≥75 | 1.079 (0.752-1.549) | *0.679* | 1.042 (0.712-1.523) | *0.834* |
| BW loss |  |  |  |  |
| <10% | 1 |  | 1 |  |
| ≥10% | 1.156 (0.805-1.661) | *0.433* | 1.156 (0.793-1.684) | *0.452* |
| BMI |  |  |  |  |
| <20 | 1 |  | 1 |  |
| ≥20 | 1.024 (0.712-1.474) | *0.897* | 1.071 (0.733-1.566) | *0.723* |
| ECOG PS score |  |  |  |  |
| 0-1 | 1 |  | 1 |  |
| 2 | 1.262 (0.818-1.947) | *0.293* | 1.360 (0.860-2.151) | *0.189* |
| Cohort |  |  |  |  |
| Cohort 1 (targeted therapy) | 0.412 (0.242-0.699) | *0.001* | 0.444 (0.250-0.790) | *0.006* |
| Cohort 2 (CTx ± ICIs) | 0.706 (0.461-1.083) | *0.111* | 0.750 (0.478-1.177) | *0.211* |
| Cohort 3 (ICIs) | 1 |  | 1 |  |

Data are presented as hazard ratio (95% confidence interval).

BMI, body mass index; BW, body weight; CTx, cytotoxic chemotherapy; ECOG, Eastern Cooperative Oncology Group; ICI, immune checkpoint inhibitor; PFS, progression-free survival; PS, performance status

**Table S5b.** Univariate and Multivariate Analysis for OS

|  | Hazard ratio (95% confidence interval) | | | |
| --- | --- | --- | --- | --- |
|  | Univariate analysis | *p*-value | Multivariate analysis | *p*-value |
| Sex |  |  |  |  |
| Male | 1 |  | 1 |  |
| Female | 0.676 (0.414-1.105) | *0.118* | 0.821 (0.483-1.396) | *0.467* |
| Age |  |  |  |  |
| <75 | 1 |  | 1 |  |
| ≥75 | 1.093 (0.700-1.709) | *0.696* | 1.166 (0.732-1.857) | *0.519* |
| BW loss |  |  |  |  |
| <10% | 1 |  | 1 |  |
| ≥10% | 1.045 (0.668-1.636) | *0.847* | 1.016 (0.638-1.620) | *0.946* |
| BMI |  |  |  |  |
| <20 | 1 |  | 1 |  |
| ≥20 | 0.684 (0.442-1.058) | *0.088* | 0.698 (0.443-1.099) | *0.121* |
| ECOG PS score |  |  |  |  |
| 0-1 | 1 |  | 1 |  |
| 2 | 1.453 (0.869-2.432) | *0.154* | 1.487 (0.870-2.540) | *0.147* |
| Cohort |  |  |  |  |
| Cohort 1 (targeted therapy) | 0.380 (0.191-0.756) | *0.006* | 0.426 (0.204-0.892) | *0.024* |
| Cohort 2 (CTx ± ICIs) | 0.731 (0.439-1.215) | *0.226* | 0.825 (0.486-1.399) | *0.475* |
| Cohort 3 (ICIs) | 1 |  | 1 |  |

Data are presented as hazard ratio (95% confidence interval).

BMI, body mass index; BW, body weight; CTx, cytotoxic chemotherapy; ECOG, Eastern Cooperative Oncology Group; ICI, immune checkpoint inhibitor; OS, overall survival; PS, performance status
