# Supplementary material for: Impact of Cachexia and First‐Line Systemic Therapy for Previously Untreated Advanced Non‐Small Cell Lung Cancer: NEJ050A
Source: J Cachexia Sarcopenia Muscle. 2024 Oct 1;15(6):2618–28. doi: 10.1002/jcsm.13606 (PMC11634491; doi:10.1002/jcsm.13606)
Supplement: Supplementary file 2 — Figure S1. Relationship between appetite‐related QOL and BW gain. Line graph showing the trends in FAACT (A/CS) score and QERD score from baseline between the groups divided by the BW gain from baseline to maximum weight up to 6 weeks later (BW gain: (a, b) ≤ 0% vs. >0%, (c, d) ≤ 2.5% vs. >2.5% and (e, f) ≤ 5.0% vs. >5.0%) in the FAS. The tables list showed the QOL scores in detail. The LS mean (SE) at 1, 3, and 6 weeks after initiating the systemic therapy and comparison between the groups using analysis of variance Abbreviations: BW, body weight, FAACT (A/CS), Functional Assessment of Anorexia/Cachexia Treatment Anorexia/Cachexia Subscale; FAS, full analysis set; LS, least squares; QERD, Questionnaire for Eating‐related Distress among Patients with Advanced Cancer; QOL, quality of life; SD, standard deviation; SE, standard error Figure S2. Time‐course change of cachexia‐related laboratory values. Line graph showing the trends in (a) haemoglobin, (b) albumin and (c) c‐reactive protein levels from baseline in each cohort in the FAS. The table lists the LS mean (SE) at 1, 3, and 6 weeks after initiating the systemic therapy and comparison among the cohorts using analysis of variance Abbreviations:CTx, cytotoxic chemotherapy; FAS, full analysis set; ICI, immune checkpoint inhibitor; LS, least squares; SE, standard error [file JCSM-15-2618-s001.pdf]

Figure S1

a. FAACT (A/CS) score (Weight gain: ≦0% vs. >0%)

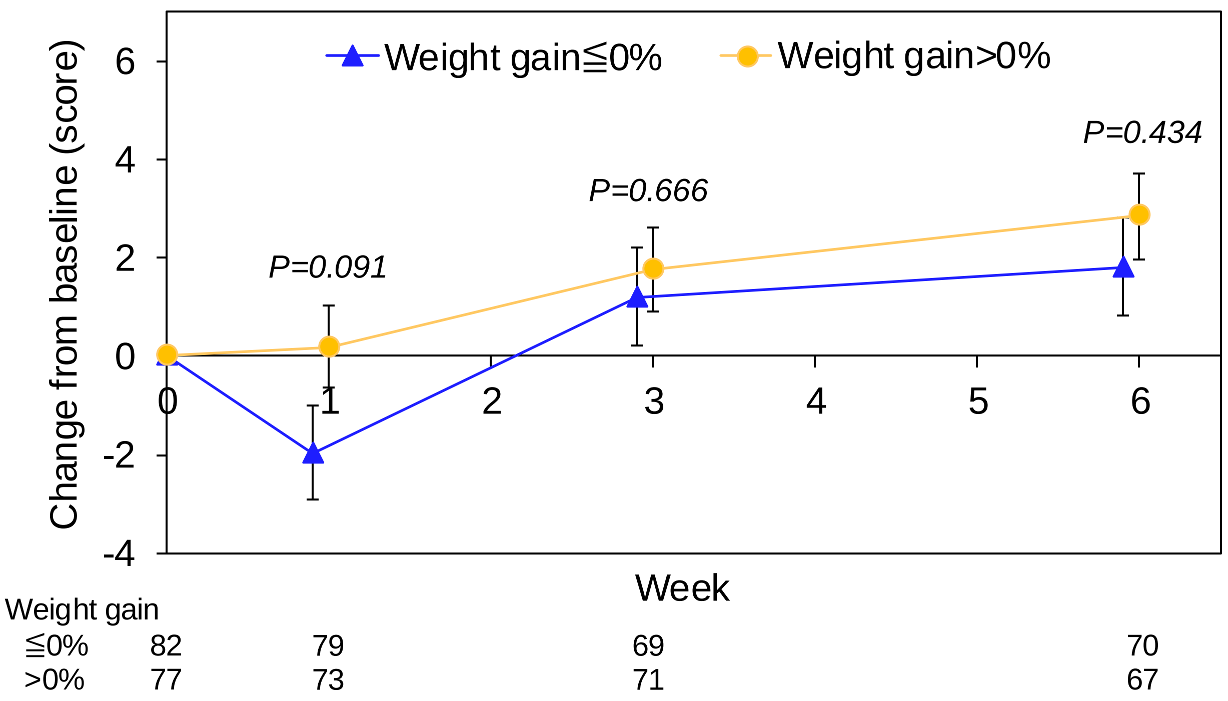

	No.	Mean	SD	SE	minimum	25th quartile	Median	75th quartile	maximum	LS mean (SE)
Week 1										
≦0%	79	-1.7	8.2	0.9	-28	-6.5	-1	3	20	-2.0 (1.0)
>0%	73	-0.2	7.0	0.8	-25	-4	1	5	12	0.2 (0.8)
Week 3										
≦0%	69	1.1	8.3	1.0	-26	-2.2	2	6	19	1.2 (1.0)
>0%	71	1.6	5.9	0.7	-15	-2	2	4	19	1.8 (0.8)
Week 6										
≦0%	70	1.6	9.9	1.2	-26	-4.5	1.5	8	28	1.8 (1.0)
>0%	67	2.5	7.1	0.9	-17	-1	2	7	20	2.8 (0.9)

b. QERD score (Weight gain: ≦0% vs. >0%)

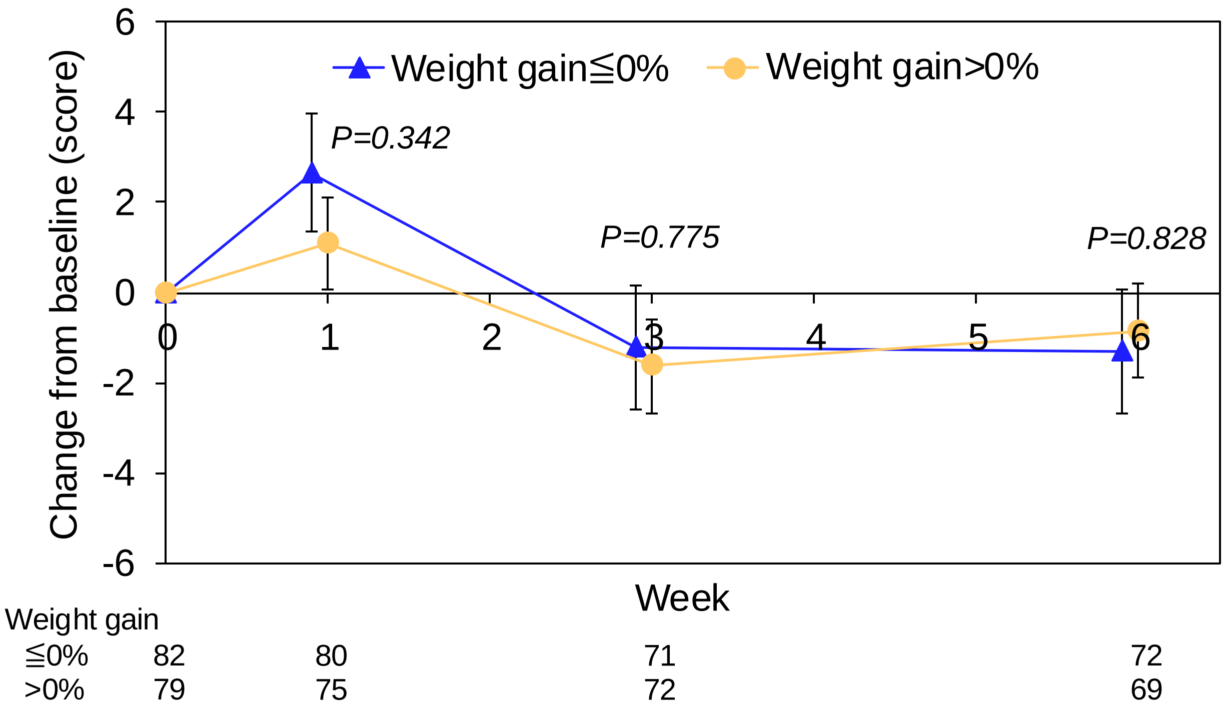

	No.	Mean	SD	SE	minimum	25th quartile	Median	75th quartile	maximum	LS mean (SE)
Week 1										
≦0%	80	2.4	12.0	1.3	-24	-4.8	0	10.3	35	2.7 (1.3)
>0%	75	1.3	8.5	1.0	-16.7	-4	0	6.5	27	1.1 (1.0)
Week 3										
≦0%	71	-0.9	12.0	1.4	-25	-8	-2	3.5	37	-1.2 (1.4)
>0%	72	-1.4	7.1	0.8	-20	-5.3	-1	1.4	18.5	-1.6 (1.0)
Week 6										
≦0%	72	-1.2	11.9	1.4	-27	-9	-1.5	7	32	-1.3 (1.4)
>0%	69	-0.7	9.4	1.1	-19	-6	0	4	40	-0.9 (1.0)

Figure S1

c. FAACT (A/CS) score (Weight gain: ≦2.5% vs. >2.5%)

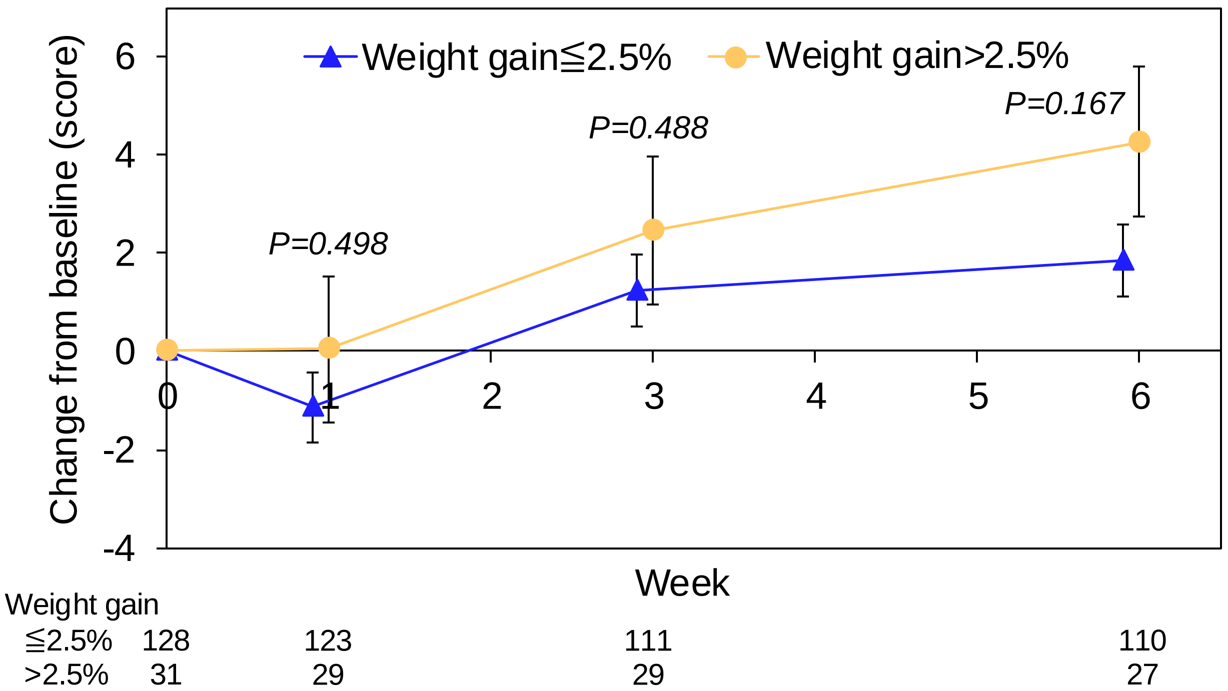

	No.	Mean	SD	SE	minimum	25th quartile	Median	75th quartile	maximum	LS mean (SE)
Week 1										
≦2.5%	123	-1.0	7.5	0.7	-28	-5.3	0	4	20	-1.1 (0.7)
>2.5%	29	-1.0	8.5	1.6	-25	-4	0	5	10	0.0 (1.5)
Week 3										
≦2.5%	111	1.1	7.6	0.7	-26	-2.1	2	5.2	19	1.3 (0.7)
>2.5%	29	2.3	5.1	0.9	-7	-1.2	2	4	14	2.5 (1.5)
Week 6										
≦2.5%	110	1.6	9.0	0.9	-26	-3	2	7.5	28	1.8 (0.7)
>2.5%	27	3.8	6.6	1.3	-10	-0.5	2	8.3	20	4.3 (1.5)

d. QERD score (Weight gain: ≦2.5% vs. >2.5%)

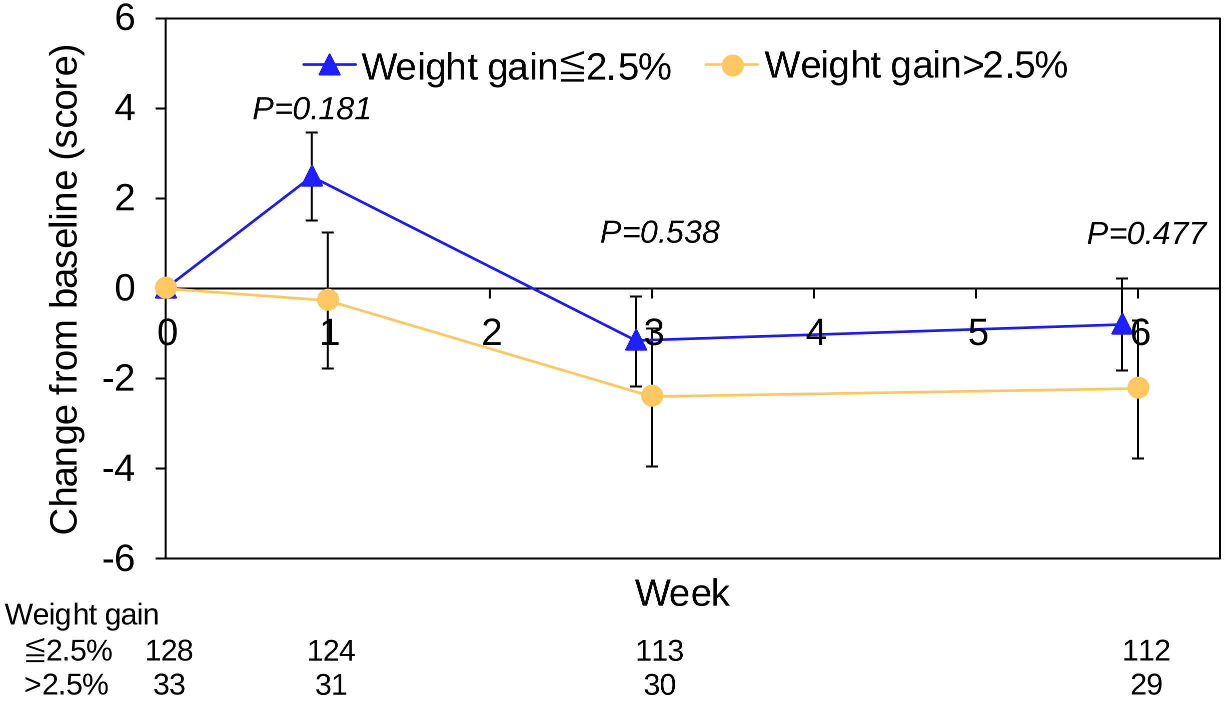

	No.	Mean	SD	SE	minimum	25th quartile	Median	75th quartile	maximum	LS mean (SE)
Week 1										
≦2.5%	124	2.4	10.9	1.0	-24	-4	0	10	35	2.5 (1.0)
>2.5%	31	-0.2	8.2	1.5	-16.7	-4	0	3	27	-0.3 (1.5)
Week 3										
≦2.5%	113	-0.8	10.5	1.0	-25	-7	-1	3	37	-1.2 (1.0)
>2.5%	30	-2.3	6.4	1.2	-14	-7.3	-0.5	0.5	18.5	-2.4 (1.5)
Week 6										
≦2.5%	112	-0.7	11.3	1.1	-27	-7	-0.5	5.3	40	-0.8 (1.0)
>2.5%	29	-2.1	8.2	1.5	-17	-8	0	3	13	-2.3 (1.5)

Figure S1

e. FAACT (A/CS) score (Weight gain: ≦5% vs. >5%)

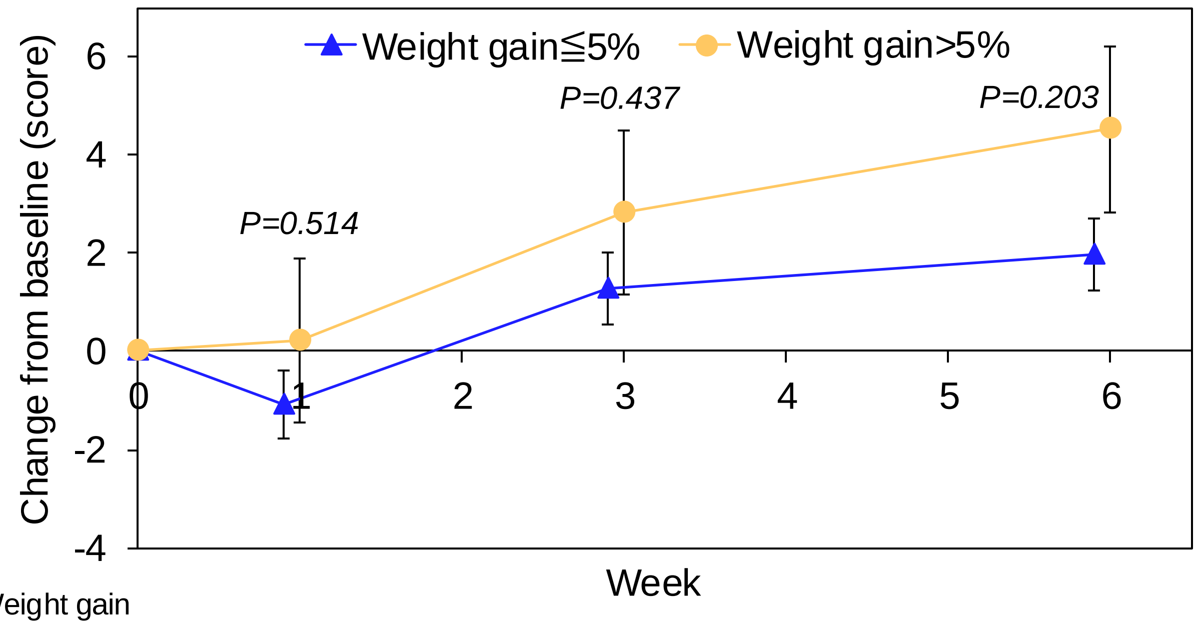

Weight gain

≦5%	138	132	120	118
>5%	21	20	20	19

	No.	Mean	SD	SE	minimum	25th quartile	Median	75th quartile	maximum	LS mean (SE)
Week 1										
≦5%	132	-1.1	7.7	0.7	-28	-5.2	0	4	20	-1.1 (0.7)
>5%	20	-0.2	7.4	1.6	-18	-4.5	1	6.3	10	0.2 (1.7)
Week 3										
≦5%	120	1.2	7.4	0.7	-26	-2	2	5	19	1.3 (0.7)
>5%	20	2.4	5.3	1.2	-7	-0.3	2	4.7	14	2.8 (1.7)
Week 6										
≦5%	118	1.7	8.8	0.8	-26	-3	2	7.5	28	2.0 (0.7)
>5%	19	4.1	7.1	1.6	-10	0.5	3	8.8	20	4.5 (1.7)

f. QERD score (Weight gain: ≦5% vs. >5%)

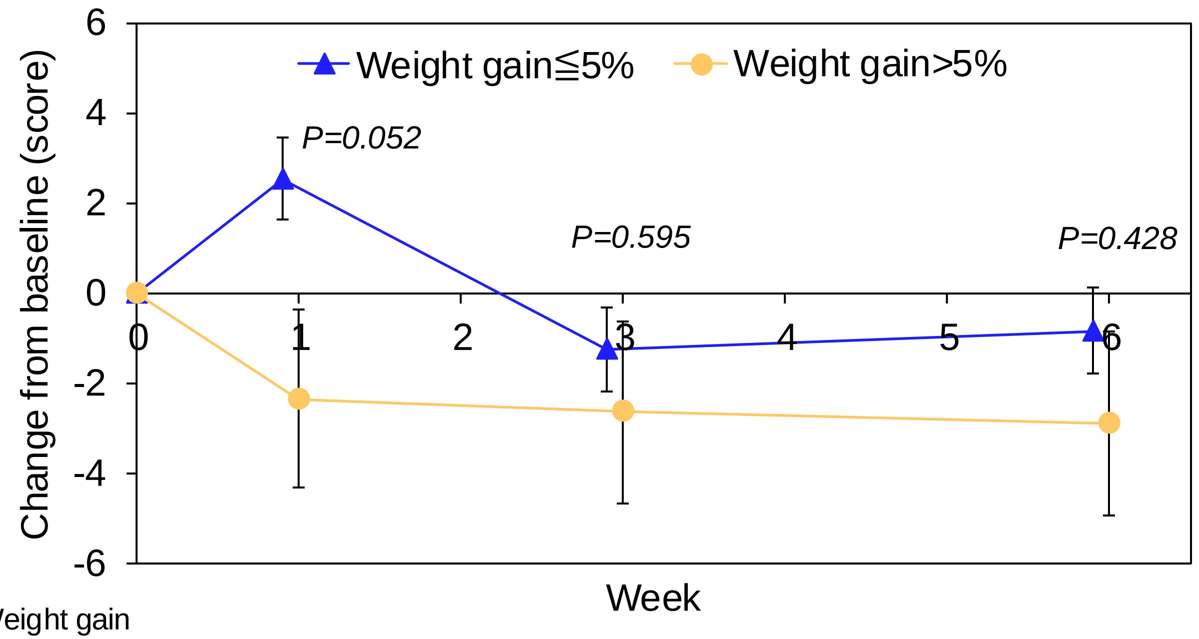

Weight gain

≦5%	140	135	124	122
>5%	21	20	19	19

	No.	Mean	SD	SE	minimum	25th quartile	Median	75th quartile	maximum	LS mean (SE)
Week 1										
≦5%	135	2.5	10.7	0.9	-24	-4	0	10	35	2.5 (0.9)
>5%	20	-2.2	7.5	1.7	-16.7	-6	0	3	10	-2.4 (2.0)
Week 3										
≦5%	124	-0.9	10.1	0.9	-25	-6.3	-1	2.3	37	-1.3 (0.9)
>5%	19	-2.5	7.5	1.7	-14	-9	-1	0.8	18.5	-2.7 (2.0)
Week 6										
≦5%	122	-0.7	11.0	1.0	-27	-7	0	5	40	-0.8 (0.9)
>5%	19	-3.0	8.9	2.0	-17	-10	-2	1.1	13	-2.9 (2.0)

Figure S2

a. Hemoglobin

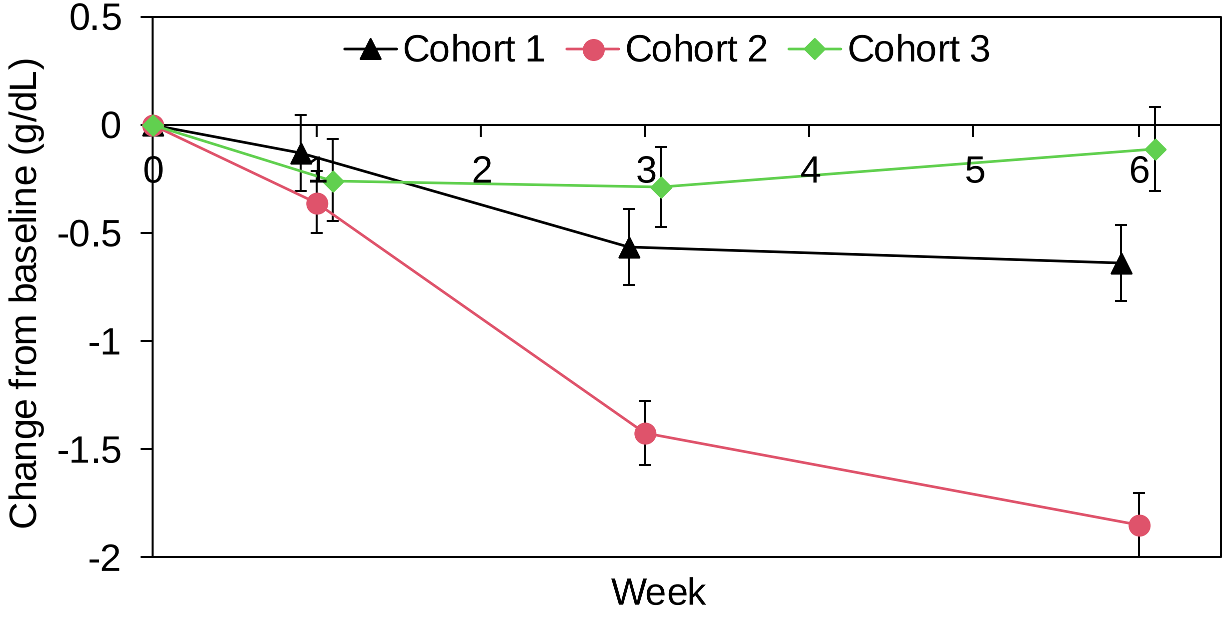

Cohort 1	41	40	41	41
Cohort 2	87	85	81	80
Cohort 3	37	34	35	30

LS mean (SE)	Cohort 1 (n=41)	Cohort 2 (n=87)	Cohort 3 (n=37)	Cohort 1 vs. Cohort 2	Cohort 1 vs. Cohort 3	Cohort 2 vs. Cohort 3
Week 1	-0.1 (0.2)	-0.4 (0.1)	-0.3 (0.2)	*P=0.341*	*P=0.621*	*P=0.705*
Week 3	-0.6 (0.2)	-1.4 (0.1)	-0.3 (0.2)	*P<0.001*	*P=0.282*	*P<0.001*
Week 6	-0.6 (0.2)	-1.8 (0.1)	-0.1 (0.2)	*P<0.001*	*P=0.050*	*P<0.001*

Cohort 1: targeted therapy, Cohort 2: CTx ± ICIs, Cohort 3: ICIs

Figure S2

b. Albumin

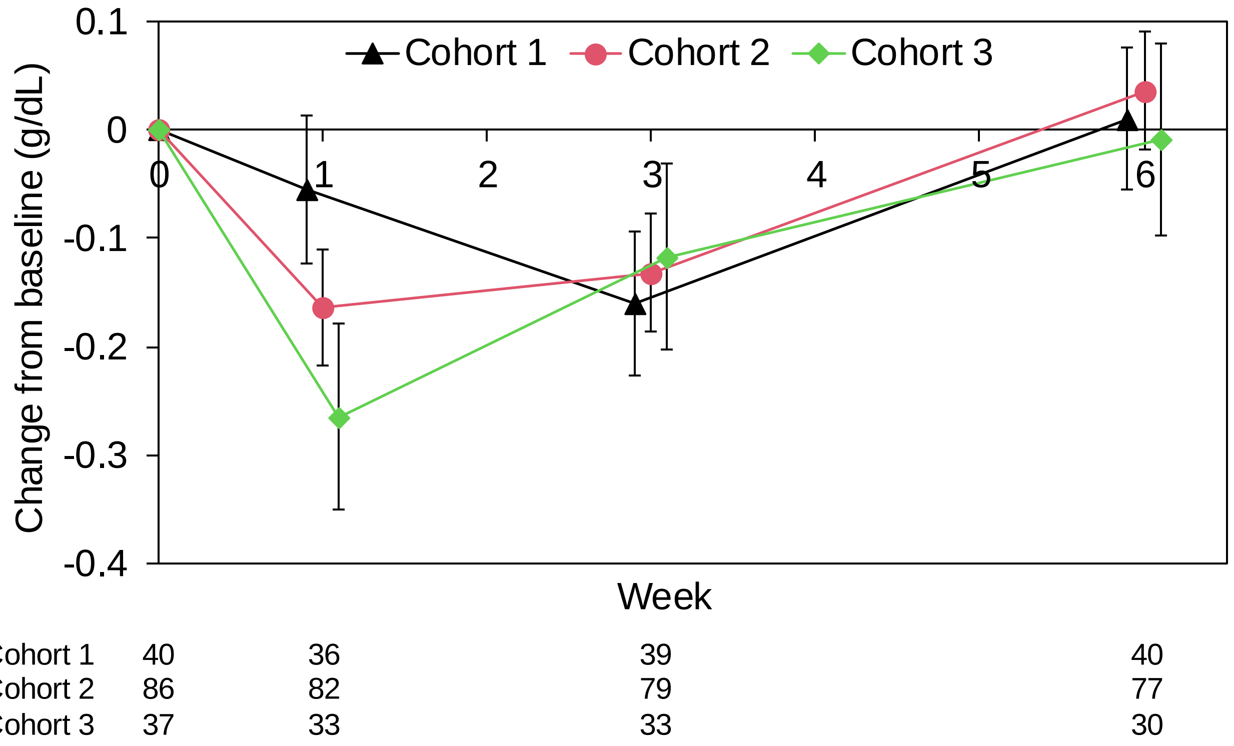

LS mean (SE)	Cohort 1 (n=41)	Cohort 2 (n=87)	Cohort 3 (n=37)	Cohort 1 vs. Cohort 2	Cohort 1 vs. Cohort 3	Cohort 2 vs. Cohort 3
Week 1	-0.05 (0.07)	-0.16 (0.05)	-0.27 (0.09)	*P=0.234*	*P=0.054*	*P=0.311*
Week 3	-0.16 (0.07)	-0.13 (0.05)	-0.12 (0.09)	*P=0.748*	*P=0.691*	*P=0.879*
Week 6	0.01 (0.07)	0.04 (0.05)	-0.01 (0.09)	*P=0.778*	*P=0.851*	*P=0.675*

Cohort 1: targeted therapy, Cohort 2: CTx ± ICIs, Cohort 3: ICIs

Figure S2

c. C-reactive protein

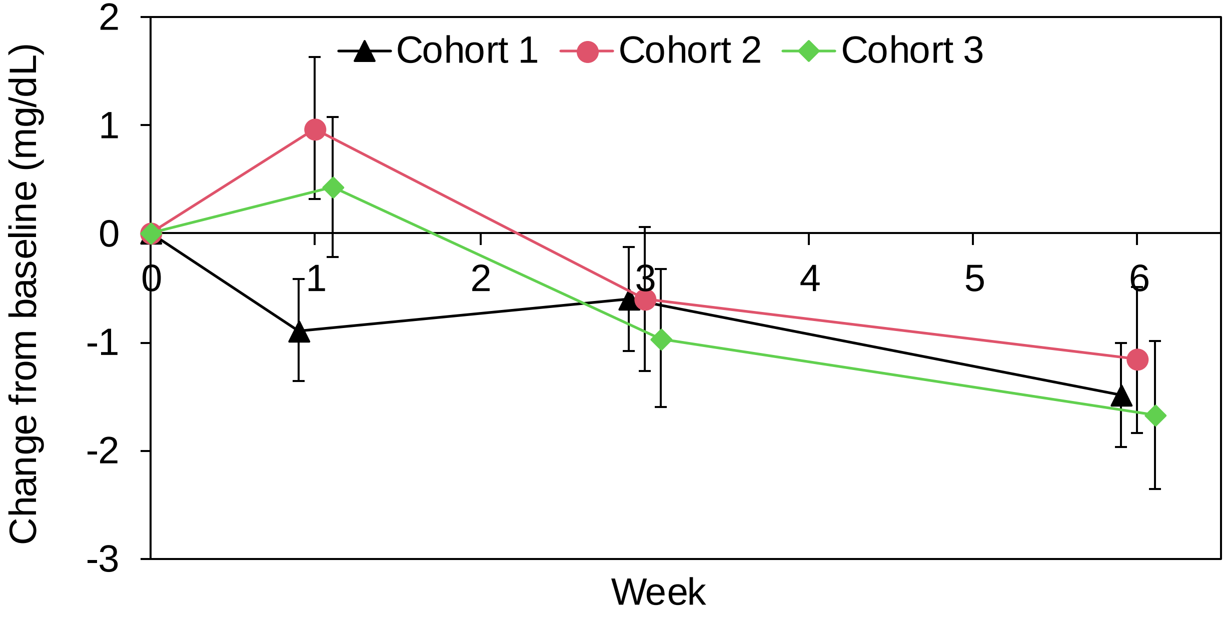

Cohort 1	41	40	40	40
Cohort 2	86	82	80	78
Cohort 3	37	34	35	30

LS mean (SE)	Cohort 1 (n=41)	Cohort 2 (n=87)	Cohort 3 (n=37)	Cohort 1 vs. Cohort 2	Cohort 1 vs. Cohort 3	Cohort 2 vs. Cohort 3
Week 1	-0.9 (0.5)	1.0 (0.7)	0.4 (0.6)	*P=0.065*	*P=0.094*	*P=0.629*
Week 3	-0.6 (0.5)	-0.6 (0.7)	-1.0 (0.6)	*P=0.996*	*P=0.639*	*P=0.749*
Week 6	-1.5 (0.5)	-1.2 (0.7)	-1.7 (0.7)	*P=0.749*	*P=0.815*	*P=0.617*

Cohort 1: targeted therapy, Cohort 2: CTx ± ICIs, Cohort 3: ICIs
